# Racial variations in sciatic nerve anatomy: A systematic review and meta-analysis

**DOI:** 10.1371/journal.pone.0344170

**Published:** 2026-03-05

**Authors:** Seid Mohammed Abdu, Hussen Abdu, Endris Seid Muhaba, Ebrahim Msaye Assefa, Gosa Mankelkl

**Affiliations:** 1 School of Biomedical Science, College of Medicine and Health Sciences, Wollo University, Dessie, Ethiopia; 2 Department of Human Anatomy, School of Medicine and Pharmacy, College of Medicine and Health Sciences, University of Rwanda, Huye, Rwanda; Menzies School of Health Research: Charles Darwin University, AUSTRALIA

## Abstract

**Background:**

The sciatic nerve (SN), the longest and largest nerve in the body, arises from the L4-S3 nerve roots and exits as a single trunk below the piriformis muscle through the greater sciatic foramen. However, variations in its anatomy are common, believed to originate from embryological development. These variations show significant racial and geographical differences, which have often been overlooked in previous review studies. Therefore, this meta-analysis aims to address this gap by systematically reviewing global data to evaluate the impact of race on sciatic nerve variations.

**Methods:**

A systematic review and meta-analysis were conducted to assess the pooled prevalence of SN variations among racial subgroups. A comprehensive literature search was performed using PubMed, Google Scholar, Hinari, and additional sources, including major anatomical journals and cross-referenced articles. Subgroup analyses by region and country were also conducted using a random-effects model. Heterogeneity was assessed with the Cochrane Q test and the I² statistic.

**Results:**

Type A, considered the normal pattern, had the highest pooled prevalence at 86%. The remaining 14% represented variations of the sciatic nerve (SN). Among these, Type B was the most common at 7%, followed by Type C and G each observed in 2% of limbs, while less frequent variations included Type Type D (1%), Type E (0%), and Type F (0% (0–1)). Racial analysis showed that SN variations occurred in 15% of Asians, 12% of Whites, and 13% of Blacks. Regarding continents, the highest prevalence was in Asia with 15%, the second highest prevalence was observed in Europe with 14%, followed by Africa with 13%, and the lowest in America with 11%. No significant differences were found among the races and continents. However, East Asia showed the highest significant prevalence, with China at 35% and Japan at 32%.

**Conclusion:**

This review revealed only modest and statistically non-significant differences in the prevalence of sciatic nerve variations across broad racial and continental groups. In contrast, substantial variation was observed at the regional level, with particularly high prevalence rates in East Asian countries, specifically China and Japan. These findings suggest that regional factors contribute more to the observed variations than racial factors.

## Introduction

The sciatic nerve (SN), the largest nerve in the human body, originates from the lumbosacral plexus (L4–S3) and typically exits below the piriformis muscle as a single trunk [1]. It usually divides into the tibial nerve (TN) and common peroneal nerve (CPN) at the apex of the popliteal fossa [2]. However, anatomical variations are common, often due to the TN and CPN developing separately during the embryological period [3].

To systematically describe these variations, particularly in relation to the piriformis muscle, Beaton and Anson introduced a classification system in 1937 [4]. Their framework, which remains the primary reference, identified six major types of SN variants. Subsequent studies have built on or modified this system, often categorizing variations into two broad groups: those where the SN exits the pelvis as a single trunk, and those where it exits pre-divided into the TN and CPN. As updated by Tomaszewski et al. [5], the variations of the SN are further sub-classified based on the relationship between its divisions and the piriformis muscle (PM). In Type A, the SN exits the pelvis undivided below the PM. Type B involves a divided SN, where the common peroneal nerve (CPN) pierces the PM and the tibial nerve (TN) passes below. In Type C, the SN is divided, with the CPN passing over the PM and the TN below. Type D describes an undivided SN piercing the PM. Type E shows a divided SN, with the CPN passing over and the TN piercing the PM. In Type F, the undivided SN exits over the PM, while in Type G, the SN divides with both the CPN and TN coursing separately below the PM. This classification provides a comprehensive anatomical framework for understanding the positional variations of the sciatic nerve in relation to the piriformis muscle.

Therefore, understanding the variable anatomy of the SN is crucial for performing safe procedures such as total hip arthroplasty (THA) and SN blocks, as well as for avoiding iatrogenic injury. Moreover, the varying relationship with the piriformis muscle can also cause piriformis syndrome due to its entrapment [3].

Previous meta-analyses by Tomaszewski et al. [5] and Frideriki Poutoglidou et al.[6] reported varying pooled prevalence rates of SN variations, ranging from 10% to 31% across different regions. Although the concept of race is both socially and biologically complex, studies have reported clear racial differences between populations, with significant distinctions observed between Chinese and Japanese on one hand, and Europeans and Americans on the other [7]. However, while these studies primarily examined anatomical variations in relation to the piriformis muscle, they did not explicitly investigate racial differences, even though they reported significant disparities among populations. To address this gap in the literature, our meta-analysis aims to systematically retrieve global data from anatomical studies and comprehensively analyze and summarize existing evidence on how race and region influences sciatic nerve variations, providing insights relevant for clinical procedures and anatomical studies.

## Methods

### 2.1. Search strategy

The Preferred Reporting Items for Systematic Reviews and Meta-Analyses (PRISMA) guidelines were followed in the reporting of this systematic review and meta-analysis [8] (S1 Checklist). Studies published from the start of each database to January 25, 2026 were included. A comprehensive literature search was conducted across multiple electronic databases, including PubMed, Google Scholar, HINARI, and a hand-search of the major anatomical journals (Annals of Anatomy, Clinical Anatomy, Journal of Anatomy, Anatomical Record, Surgical and Radiological Anatomy, Folia Morphologica, European Journal of Anatomy, Anatomical Science International, Anatomy and Cell Biology, Morphologie) was performed. Three steps were involved in the search strategy. The first step involved finding pertinent Medical Subject Headings (MeSH) in the literature. Complete searches were carried out in the aforementioned databases during the second phase. In the third stage, university websites and the bibliographies of pertinent studies were examined for the existence of studies that qualified. In the third stage, university websites, major anatomical journals and the bibliographies of relevant studies were examined to identify additional eligible studies. Various MeSH terms and keywords were combined using Boolean operators (OR, AND) to search the databases.

The following search terms were used: “Variation”[Title/Abstract] OR “Anatomy”[Title/Abstract] OR “Position”[Title/Abstract] OR “Relation”[Title/Abstract] OR “Interrelation”[Title/Abstract] OR “Course”[Title/Abstract] OR “Division”[Title/Abstract] OR “Observation”[Title/Abstract] OR “Morphology”[Title/Abstract] OR “Variability”[Title/Abstract] OR “Topography”[Title/Abstract] OR “Variant”[Title/Abstract] OR “Anatomical variation”[Title/Abstract] OR “anatomical position”[Title/Abstract] AND “Sciatic nerve”[Title/Abstract] OR “nerve sciatic”[Title/Abstract] OR “sciatic nerves”[Title/Abstract] OR “nerves sciatic”[Title/Abstract]. Filters applied: English language, free abstract text availability, and publication date up to January 25, 2026 (S1 Appendix).

### 2.2. Criteria for considering studies for the review

#### Inclusion criteria.

The design of the study was based on observational studies comprises of pertinent description for our analysis, including individuals of any age in the population. The research was limited to cadaveric studies carried out worldwide, and only at least abstract articles written in English were considered. Moreover, this systematic review and meta-analysis included studies published from the inception of each database up to January 25, 2026. All studies that reported extractable anatomical data on the branching patterns of the SN were included. Furthermore, in this review, racial categorization was extracted based on the description provided in the original articles. Where available, we used self-identified race. If the original authors explicitly described the race of study participants, we reported it as described. In cases where neither of these was specified, racial classification was inferred based on the dominant racial group of the study’s geographic location.

#### Exclusion criteria.

Case reports, case series studies, letters to the editor, image based studies and any other research lacking the necessary data to report anatomical data on the branching patterns of the SN was excluded.

### 2.3. Data extraction and quality assessment

The articles’ eligibility was evaluated independently by two investigators (SMA and HA). The same two writers used Microsoft Excel to separately extract data. Author name, race, countries, continent, SN branching pattern related to piriformis, sample size, number of total variation were all included in the data extraction sheet. By reviewing the article and thorough discussion, disagreements amongst investigators were settled. Titles and abstracts of the identified articles were examined in order to find studies on SN branching pattern. Articles that the title and abstract deemed relevant were vetted for complete eligibility. Based on the cadaver results and presentation of the studies, the methodological quality of the included studies was evaluated using Anatomical Quality Assurance (AQUA) checklist [9].

### 2.4. Outcome of Interest

The main goal of this systematic review and meta-analysis was to determine global racial variations in sciatic nerve anatomy, as reported either as a percentage or as the number of cases (n) out of the total number of cadaver assessed (N)

### 2.5. Statistical Analysis

Using the random-effects inverse-variance method, the pooled prevalence of multi-categorical racial variations in sciatic nerve anatomy was determined, along with the corresponding 95% confidence intervals (CI). The pooled prevalence of the sciatic nerve racial variation was analyzed using MetaXL version 5.3 (EpiGear International Pty Ltd., Wilston, Queensland, Australia). Heterogeneity among studies was evaluated using Cochran’s Q test and the I² statistic. Preplanned subgroup analyses were conducted according to predefined criteria. Non-overlapping 95% CIs were interpreted as evidence of statistically significant differences between subgroups, whereas overlapping CIs indicated no significant difference [10]. Potential publication bias was examined using Doi plots and the Luis Furuya-Kanamori (LFK) index, which are analytical tools well-suited for anatomical meta-analyses implemented in MetaXL.

## Results

**Fig 1:**
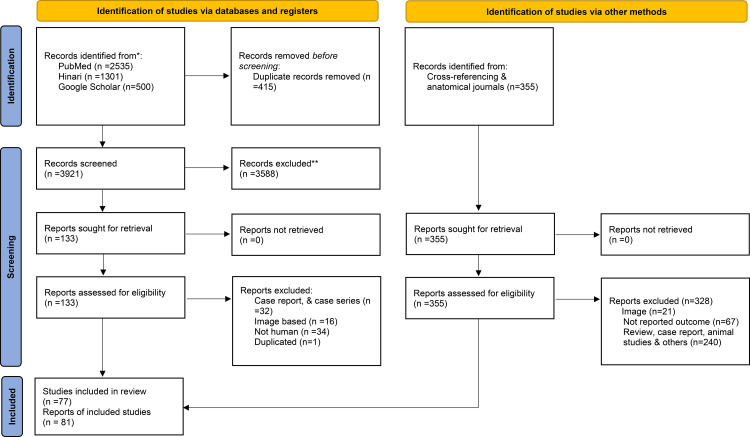
PRISMA flowchart summarizing the selection process.

In this systematic review and meta-analysis, a total of 4,336 records were retrieved from PubMed, Hinari, and Google Scholar. After the removal of 415 duplicates, 3,921 records remained for title and abstract screening. Of these, 3,588 were excluded based on relevance, and 133 full-text articles were sought for retrieval. All 133 articles were successfully retrieved and screened for eligibility, resulting 50 eligible studies. Among these, one study from South Africa and another from Brazil each provided three separate reports, resulting in a total of 54 individual reports. Additionally, 355 articles were identified through a secondary search, including reference lists, gray literature, and hand-on searches of anatomy journals, of which 27 studies met the eligibility criteria and were included. This brought the total number of included reports to 81. The detailed study selection process is illustrated in Fig 1.

### Study Characteristics

Characteristics of the included studies are summarized in S1 Table. A total of 81 reports, (11,089 total limbs) were included in this systematic review and meta-analysis [4,7,11–72,73–79]. The reports were published between 1897 and January, 2026 and involved populations of diverse geographical and racial backgrounds. Of the 81 reports, 38 were conducted in Asia, 17 in Africa, 12 in Europe, and 14 in North and South America. Notably, 23 reports originated from India, and 8 from the USA. Regarding participant race, 33 reports involved Asian participants, 29 involved White participants, and 19 involved Black participants.

### Risk of bias Assessment using AQUA checklist

**Fig 2:**
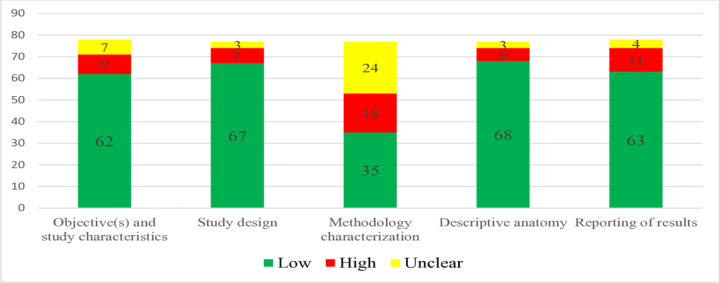
Risk of bias assessment of included studies using the AQUA checklist across five.

The AQUA checklist revealed that most anatomical studies maintained a low risk of bias in study design, objectives, descriptive anatomy, and results reporting. However, methodology characterization emerged as a critical area needing improvement, as nearly 55% of the studies were rated as unclear or high risk in this domain (Fig 2)

### The Pooled prevalence of sciatic nerve variation

The pooled prevalence of SN variation was 14% (95% CI: 11–16). Among these atypical variations, the most common type was Type B, accounting for 53%, followed by Type C 18%, Type G 13%, and Type D 7%. The remaining Types E, F, and other variation types each accounted for 3%. Significant heterogeneity was observed across all analyses, with I² values greater than 90% and P < 0.001.

Among all evaluated limbs, our review reveals that Type A variations, considered the normal branching pattern of the SN where the nerve passes undivided under the PM, are the most commonly observed, with a pooled prevalence of 86% (95% CI: 84–89). Type B, in which the SN bifurcates in the pelvis with the common peroneal nerve (CPN) piercing the PM and the tibial nerve (TN) coursing below, had a prevalence of 7% per total limbs. The third most common variations, Types C and G, were each observed in 2% of limbs. The rarer variations, D, E, and F were also observed, and are summarized in Table 1.

**Table 1:** Patterns of SN variation according to Beaton and Anson’s classification.

Types of SN variation per total limbs		N^o^ of study	N^o^ limbs	N^o^ limbs with variations	Prevalence CI 95%	I^2^	p-value	LFK-index
Type A		81	11089	9363	86(84–89)	99%	<0.001	−0.76
Type B		69	9744	1105	7(5– 9)	93%	<0.001	−1.62
Type C		69	9314	154	2(2–2)	53%	<0.001	2.54
Type D		69	9364	63	1(1–1)	47%	<0.001	288
Type E		69	9364	18	0	0.0%	0.66	3.12
Type F		69	9364	39	0(0-1)	56%	<0.001	2.90
Type G		18	9364	121	2(1–2)	82%	<0.001	3.75
Overall variation		81	11089	1705	14(11–16)	92%	<0.001	0.75
Among Overall variation			N^o^ limbs with variation types					
	Type B	67	979		53(43-62)	90%	<0.001	−6.23
	Type C	67	155		18(11–25)	90%	<0.001	3.17
	Type D	67	30		7(3–13)	90%	<0.001	2.67
	Type E	67	4		3(1– 8)	90%	<0.001	3.95
	Type F	67	39		3(1–8)	90%	<0.001	3.52
	Type G	67	120		13(7–19)	90%	<0.001	4.45

From the available data on the racial variation of the SN, it was found that 16% of SN variation was observed in individuals of Asian origin. The pooled prevalence of SN variation was 12% for the white race and 13% for the black race. Subgroup analysis was also performed by continent and country. The highest prevalence of SN variation was recorded in Asia at 15% (10–20), while the lowest was in America at 11%. The second highest prevalence was observed in Europe at 14%, followed by Africa at 13%.

Additionally, the pooled prevalence was further analyzed among countries, with the highest prevalence recorded in East Asia, specifically in China and Japan, at 35% (19–41) and 32% (24–39), respectively (Table 2).

**Table 2:** Subgroup analysis of SN by race, countries and geographic region (continent).

Racial variation of SN						
Race/ Ethnicity	N^o^ of studies	N^o^ limb	Prevalence CI 95%	I^2^	P-value	LFK-index
White	29	5760	12(10–15)	88%	<0.001	0.75
Asian origin	33	3695	15(11–20)	93%	<0.001	0.75
Black	19	1634	13 (9 –18)	85%	<0.001	0.75
**SN variation by Countries**						
Country	N^o^ of studies	N^o^ limb	Prevalence CI 95%	I^2^	P-value	LFK-index
Ethiopia	3	142	10(5 –15)	0	0.9	0.63
Nigeria	4	488	9(4 –14)	73	0.01	0.63
South Africa	5	762	10(4 –18)	88	<0.001	0.63
China	3	738	35(29 –41)	62%	0.07	0.63
Japan	3	994	32(24 –39)	80	0.01	0.63
Poland	3	266	19(6-32)	82%	<0.001	0.63
USA	8	3288	11(9 –13)	60%	0.01	0.63
Turkey	5	672	15(1-32)	96%	<0.001	0.63
Nepal	4	188	16(9 –25)	57%	0.07	0.63
Brazil	6	220	12(6 –18)	38%	0.15	0.63
India	23	1775	11(8 –15)	79%	0.01	0.63
**SN variation in Continent**						
Africa	17	1802	13(9 –18)	87%	<0.001	0.75
Asia	38	4367	15(10 –20)	94%	<0.001	0.75
Europe	12	1412	14(9 –19)	85%	<0.001	0.75
America	14	3508	11(9 –13)	50%	<0.001	0.75

**Fig 3:**
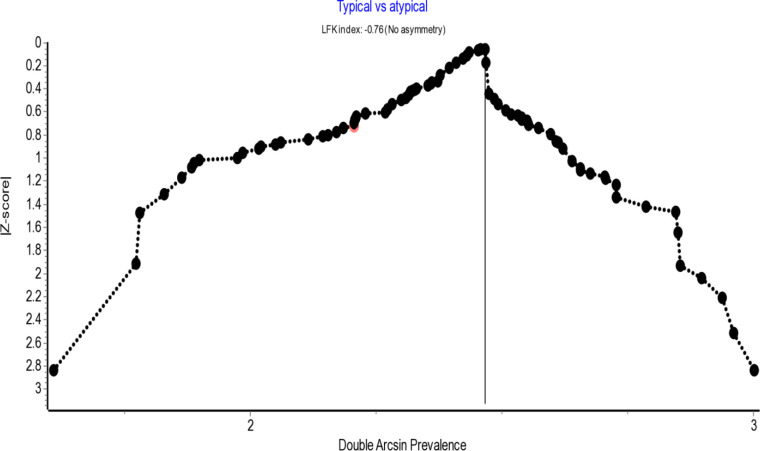
Doi plot analysis showing no publication bias in typical sciatic nerve presentations.

**Fig 4:**
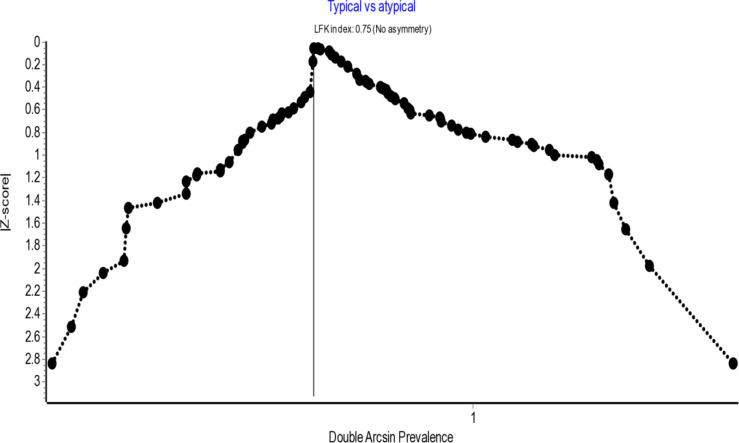
Doi plot assessing publication bias for overall sciatic nerve variations.

Assessment of publication bias using the LFK index derived from the Doi plot analysis revealed no asymmetry for either the typical presentations (LFK = −0.76) or the overall SN variations (LFK = 0.75), as shown in Fig 3 and Fig 4, respectively. The LFK indices for other variations including atypical SN types, racial variations, and analyses across different subgroups also indicated no, minor or major asymmetry, as summarized in Tables 1 and 2. Furthermore, sensitivity analyses excluding individual studies demonstrated that the pooled prevalence estimates remained within the 95% confidence intervals of the overall analysis, indicating that the findings are robust and not substantially influenced by any single report.

## Discussion

The SN is known for its anatomical variations, particularly in its exit from the pelvis and its relationship with the PM. While such variations have been reviewed by Tomaszewski et al. [5] and Frideriki P [6], no comprehensive reviews assess the influence of population origin and race. Therefore, this review aimed to provide an in-depth analysis of the SN variations by synthesizing data from all available literature to assess the influence of population origin and race. Variations in SN anatomy have significant clinical implications, as they can affecting the surgical outcomes, anesthetic procedures, and the diagnosis of neuropathies. The other pivotal aspects of this review was also the comparison of our findings with in widely used Western-based anatomy textbooks, which have long served as the gold standard for anatomical education and clinical reference. However, they often predominantly reflect anatomical data from Western populations, which may not accurately represent the anatomical variations seen in other racial groups. By exploring discrepancies between textbook descriptions and our findings, we point-out the need for a broader representation of anatomical data in educational resources. This can enhance the accuracy and relevance of anatomical knowledge for healthcare providers worldwide.

Our research reveals that Type A variations considered the normal branching pattern of the SN, where the nerve passes undivided under the PM are the most commonly observed. This typical pattern was found in approximately 86% of the population analyzed. This finding aligns with other review studies conducted by Tomaszewski et al. [5], who reported a prevalence of 85.2%, and Frideriki P. [6], who found it to be 90%. Similarly, Type A variations have been reported in approximately 85–90% of individuals according to Clinically Oriented Anatomy [80], and Netter’s Atlas of Human Anatomy [81] and in 85–88% as noted in Gray’s Anatomy [82].

The most common non-normal variation was Type B, where the SN divides in the pelvis, with the CPN piercing the PM and the TN coursing deep to the PM, with a prevalence of 7%. The second most common variation was Type G, with a prevalence of 2%, where the SN divides in the pelvis and both the CPN and TN course separately below the PM.

According to our analysis on the racial variation of the SN, we found that 15% of SN variations were observed in individuals of Asian origin. This is slightly higher compared to the pooled prevalence of SN variations, which was 12% for individuals of white race and 13% for individuals of black race. These findings suggest that while there are some differences in the prevalence of SN variations among different racial groups, the variations are not significantly pronounced, and it is a relatively common occurrence across all studied racial groups, with only modest differences in prevalence. These insights are crucial for clinical procedures and anatomical studies, as they highlight the importance of considering individual anatomical variability rather than relying solely on racial categorization. The lack of significant racial differences observed in our analysis underscores the need for personalized approaches in medical practice and research.

However, our analysis recorded the highest prevalence of SN variations in East Asia, specifically in China and Japan, with 35% and 32% respectively. These regional variations suggest that while racial differences may not be pronounced on a broader scale, certain populations, such as those in East Asia, exhibit a notably higher prevalence of SN variations. This emphasizes the importance of region-specific studies and tailored clinical approaches in understanding and addressing anatomical variations. It is important to note, however, that subgroup analyses with fewer than five studies are considered less reliable. In this case, the higher prevalence observed in China and Japan is based on only three studies each, making these estimates highly uncertain.

Awareness of SN anatomical variations is critical for preventing nerve damage during surgical interventions, especially total hip arthroplasty [3,6,38,56]. The SN is particularly prone to traction-related injuries during this procedure [28,46], and variations in its courses in relation to the PM can further elevate the risk of intraoperative trauma, either from incorrectly placed surgical retractors or from direct injury when performing a tenotomy of the PM [49]. Among these, the CPN is especially at risk in anatomical types B and C. It may sustain traction injury during hip dislocation or when lengthening the limb. Moreover, SN damage can occur in the aftermath of a traumatic posterior hip dislocation. Type B variations, in particularly, are associated with increased vulnerability during hip arthroscopy procedures [6,49].

Understanding these anatomical variants is also important for performing effective SN blocks. In cases of high nerve bifurcation, as seen in types B and C, there is a significant chance of anesthetizing only the CPN or the TN, rather than the entire SN, leading to incomplete nerve blockade [83]. Awareness of these variations should therefore inform surgical and anesthetic practice. Preoperative imaging, such as MRI, can help identify high bifurcation or variant courses of the SN. During procedures like hip arthroplasty or arthroscopy, special attention must be paid to avoid excessive traction on the CPN in types B and C, and to position retractors carefully. When performing piriformis tenotomy, dissection should be minimized near the course of the nerve to prevent direct trauma. Similarly, SN blocks should be adjusted to target both branches in patients with high division patterns to ensure effective anesthesia.

Additionally, these variations may play a role in piriformis syndrome, a condition where the SN is compressed outside the pelvis at the level of the hip. This syndrome is reported in around 6% of individuals with sciatica and is characterized by pain in the buttock that may radiate down the back of the thigh and sometimes extend below the knee. Symptoms typically worsen with movements like hip flexion, adduction, and internal rotation. According to Pećina, the type B variant where the CPN passes through the PM is most commonly linked to piriformis syndrome, particularly when the nerve travels between the muscle’s tendinous portions [30,41].

A significant limitation of our study is the high heterogeneity observed among the reviewed studies. This variability could be attributed to the diverse methodologies, classification criteria, and sample sizes used, which likely contributed to the observed heterogeneity and made it challenging to draw definitive conclusions. However, as highlighted by Henry et al. (2016) [84], high heterogeneity is a common and inherent feature of anatomical meta-analyses. They recommended the use of a random-effects model to account for such among report variabilities. In line with this recommendation, we employed a random-effects model for all analyses to enhance the validity and generalizability of our findings. The inconsistency in race classification systems introduces an additional layer of complexity, making it difficult to standardize findings across different research works. In this review, if race was reported as self-identified, we used that classification. If self-identified race was not available, we relied on the racial classification reported by the study authors. When neither was provided, we inferred race based on the geographically dominant population in the region where the study was conducted. However, it is important to acknowledge that the concept of race is both socially and biologically complex, and such classifications may not fully capture the nuanced variations relevant to anatomical differences. Moreover, without access to certain subscription databases (e.g., Embase, Scopus), our search may have missed relevant studies, though we mitigated this by cross-referencing extensively.

### Limitations of the review

Our inclusion criteria were limited solely to English-language studies, as it could result in the omission of valuable study data in languages other than English. This limitation has the potential to impact the comprehensiveness and generalizability of our findings.

## Conclusion

In conclusion, this systematic review and meta-analysis highlights that while Type A remains the predominant morphological pattern of the SN, notable variations exist, particularly across different regions. Our findings suggest that regional factors, especially within East Asia, may influence SN anatomical variations more significantly than race, which did not show consistent patterns of variation. However, it is important to note that the racial classifications used in the included studies were often based on self-reporting, author-defined categories, or geographic inference, which may not accurately reflect underlying genetic or anatomical diversity. The socially and biologically complex nature of race underscores the limitations of using it as a proxy for anatomical differences.

These insights call for the adoption of standardized, more precise, and inclusive classification systems in future anatomical research. Finally, understanding these variations has direct clinical relevance, particularly for surgeons, anesthesiologists, and neurologists, who rely on detailed knowledge of nerve anatomy to avoid iatrogenic injury and improve patient outcomes.

## Supporting information

S1 Dataset Minimal data set. (XLSX)

S1 Appendix Search strategies. (DOCX)

S1 Table Supplementary table 1. (DOCX)

S1 Checklist PRISMA checklist. (DOCX)
