# Supplementary material for: Racial variations in sciatic nerve anatomy: A systematic review and meta-analysis
Source: PLoS One. 2026 Mar 5;21(3):e0344170. doi: 10.1371/journal.pone.0344170 (PMC12962491; doi:10.1371/journal.pone.0344170)
Supplement: S1 Appendix — (DOCX) [file pone.0344170.s002.docx]

**Google Scholar**

("sciatic nerve" OR "sciatic nerves") AND ("anatomical variation" OR "anatomy" OR "position" OR "relation" OR "interrelation" OR "course" OR "division" OR "observation" OR "morphology" OR "variability" OR "topography" OR "variant" OR "variation")=

**Total retrieved Article (500)**

**Hinari**

((Variation) OR (Anatomy) OR (Relation) OR (Interrelation) OR (Position) OR (Course) OR (Division) OR (Topography) OR (Variant) OR ("Anatomical variation")) AND (("Sciatic nerve") OR ("nerve sciatic") OR ("sciatic nerves") OR ("nerves sciatic")), filter Journal Article, Custom Dates, anatomy & physiology, medicine, humans, English

**Total retrieved Article (1491)**

**Note:** Although we initially retrieved 1,491 articles, only 1,301 were successfully imported. This discrepancy occurred because the Hinari system counts articles as 'saved' even before the actual saving process is completed. For example, while attempting to save articles numbered 1 to 10 in first page, the system displayed a total of 10 saved articles; however, article number 11 was already marked as saved even though we hadn’t saved it yet. When we unsaved it, the total count dropped from 10 to 9.

**PubMed**

|  | (((((((((((((((Variation[Title/Abstract]) OR (Anatomy[Title/Abstract])) OR (Position[Title/Abstract])) OR (Relation[Title/Abstract])) OR (Interrelation[Title/Abstract])) OR (Course[Title/Abstract])) OR (Division[Title/Abstract])) OR (Observation[Title/Abstract])) OR (Observation[Title/Abstract])) OR (Variability[Title/Abstract])) OR (Topography[Title/Abstract])) OR (Variant[Title/Abstract])) OR ("Anatomical variation"[Title/Abstract])) OR ("anatomical position"[Title/Abstract])) OR ("Anatomical location"[Title/Abstract]) AND ((fha[Filter]) AND (english[Filter]))) AND (((("Sciatic nerve"[Title/Abstract]) OR ("nerve sciatic"[Title/Abstract])) OR ("sciatic nerves"[Title/Abstract])) OR ("nerves sciatic"[Title/Abstract]) AND ((fha[Filter]) AND (english[Filter]))) | 2535 |
| --- | --- | --- |
|  | ((("Sciatic nerve"[Title/Abstract]) OR ("nerve sciatic"[Title/Abstract])) OR ("sciatic nerves"[Title/Abstract])) OR ("nerves sciatic"[Title/Abstract]) | 23,286 |
|  | ((((((((((((((Variation[Title/Abstract]) OR (Anatomy[Title/Abstract])) OR (Position[Title/Abstract])) OR (Relation[Title/Abstract])) OR (Interrelation[Title/Abstract])) OR (Course[Title/Abstract])) OR (Division[Title/Abstract])) OR (Observation[Title/Abstract])) OR (Observation[Title/Abstract])) OR (Variability[Title/Abstract])) OR (Topography[Title/Abstract])) OR (Variant[Title/Abstract])) OR ("Anatomical variation"[Title/Abstract])) OR ("anatomical position"[Title/Abstract])) OR ("Anatomical location"[Title/Abstract]) | 3,077,587 |
