# Supplementary material for: Racial variations in sciatic nerve anatomy: A systematic review and meta-analysis
Source: PLoS One. 2026 Mar 5;21(3):e0344170. doi: 10.1371/journal.pone.0344170 (PMC12962491; doi:10.1371/journal.pone.0344170)
Supplement: S1 Table — (DOCX) [file pone.0344170.s003.docx]

Supplementary Table 1. Characteristics of included studies

| First author (et al.) | Country | Race / Origin | Total legs |
| --- | --- | --- | --- |
| Berihu et al. | Ethiopia | Black | 56 |
| Kiros et al. | Ethiopia | Black | 36 |
| Mengistu et al. | Ethiopia | Black | 50 |
| Atoni et al. | Nigeria | Black | 56 |
| Mbaka et al. | Nigeria | Black | 98 |
| Chukwuanukwu et al. | Nigeria | Black | 52 |
| Amasiatu VC et al | Nigeria | Black | 282 |
| Habumuremyi | Rwanda | Black | 22 |
| Ogeng’o et al. | Kenya | Black | 164 |
| Kukiriza et al. | Uganda | Black | 80 |
| Kegoye ES et al | Uganda | Black | 124 |
| Bergsteedt et al. | South Africa | Black | 84 |
| Bergsteedt et al. | South Africa | Black | 338 |
| Bergsteedt et al. | South Africa | White | 232 |
| Bergsteedt et al. | South Africa | Black | 15 |
| Bergsteedt et al. | South Africa | Black | 39 |
| Ndiaye et al. | Senegal | Black | 20 |
| Ming‑Tzu et al. | China | Asian | 430 |
| Liu et al. | China | Asian | 140 |
| Lee et al. | Taiwan | Asian | 168 |
| Kubota et al. | Japan | Asian | 38 |
| Chiba et al. | Japan | Asian | 514 |
| Chiba et al. | Japan | Asian | 442 |
| Pećina et al. | Croatia | White | 130 |
| Ugrenović et al. | Serbia | White | 200 |
| Nizankowski et al. | Poland | White | 200 |
| Okraszewska et al. | Poland | White | 36 |
| Haładaj et al. | Poland | White | 30 |
| Bardeen et al. | USA | White | 246 |
| Trotter et al. | USA | White | 464 |
| Beaton et al. | USA | White | 240 |
| Beaton et al. | USA | White | 120 |
| Georgiadis et al. | USA | White | 42 |
| Benzon et al. | USA | White | 66 |
| Anson et al. | USA | White | 2008 |
| Lewis et al. | USA | White | 102 |
| Paterson et al. | Scotland | White | 23 |
| Parsons et al. | England | White | 138 |
| Parsons et al. | UK & Ireland | White | 138 |
| Kurtoglu et al. | Turkey | White | 50 |
| Divizyon et al. | Turkey | White | 50 |
| Sulak et al. | Turkey | White | 400 |
| Sinirin et al. | Turkey | White | 52 |
| Aydın Kabakcı et al. | Turkey | White | 120 |
| Natsis et al. | Greece | White | 294 |
| Pokorný et al. | Czech Republic | White | 51 |
| Pokorný et al. | Czech Republic | White | 91 |
| Ugrenović et al. | Bosnia | White | 200 |
| Indrekvam et al. | Norway | White | 19 |
| Vicente et al. | Brazil | White | 40 |
| Gomes et al. | Brazil | White | 16 |
| Gomes et al. | Brazil | Black | 12 |
| Gomes et al. | Brazil | Brown | 12 |
| Brooks et al. | Brazil | White | 50 |
| Machado et al. | Brazil | White | 100 |
| Misra et al. | India | Asian | 300 |
| Kumar et al. | India | Asian | 50 |
| Patel et al. | India | Asian | 83 |
| Sabnis et al. | India | Asian | 140 |
| Prathiba et al. | India | Asian | 100 |
| Adibatti et al. | India | Asian | 50 |
| Ansari et al. | India | Asian | 30 |
| Anithamol et al. | India | Asian | 26 |
| Deepthi et al. | India | Asian | 100 |
| Shinde et al. | India | Asian | 48 |
| Kumari et al. | India | Asian | 52 |
| Pal et al. | India | Asian | 84 |
| Wahengbam et al. | India | Asian | 60 |
| Gangulappa et al. | India | Asian | 50 |
| Kumar et al. | India | Asian | 30 |
| Philip et al. | India | Asian | 34 |
| Pitta et al. | India | Asian | 80 |
| Prakash et al. | India | Asian | 86 |
| Sharma et al. | India | Asian | 42 |
| Badaam et al. | India | Asian | 60 |
| Jkimsu et al. | India | Asian | 90 |
| Ghosh D. et al | India | Asian | 106 |
| Jyothi J. et al | India | Asian | 42 |
| Jha et al. | Nepal | Asian | 40 |
| Pandit et al. | Nepal | Asian | 50 |
| Yadav SK et al. | Nepal | Asian | 50 |
| Yadav SK et al. | Nepal | Asian | 48 |
