# Supplementary material for: Racial variations in sciatic nerve anatomy: A systematic review and meta-analysis
Source: PLoS One. 2026 Mar 5;21(3):e0344170. doi: 10.1371/journal.pone.0344170 (PMC12962491; doi:10.1371/journal.pone.0344170)
Supplement: Attachment — Submitted filename: Response to Reviewers.docx [file pone.0344170.s007.docx]

**Rebuttal letter**

Dear editor, and reviewers.

We would like to thank you for giving us the opportunity to submit a revised version of our manuscript titled “Racial Variations in Sciatic Nerve Anatomy: A Systematic Review and Meta-Analysis”. We are too grateful to you and the reviewers for taking the time and effort to read our manuscript and offer insightful comments. Based on these comments and suggestions, we have made careful modifications to the original manuscript. Your informed and helpful feedback helped us think of potential changes for the current version. We believe the manuscript has improved and hope that it meets your high standards. The authors welcome further constructive comments, if any. Please see below for a point-by-point response to comments and concerns. Comments are shown in black, followed by our answers and responses in dark blue. The changes are shown in the marked copy of the manuscript (highlighted in blue).

Best regards,

**Academic Editor’s Comment**

Dear Dr. Abdu,

Thank you for submitting your manuscript to PLOS ONE. After careful consideration, we feel that it has merit but does not fully meet PLOS ONE’s publication criteria as it currently stands. Therefore, we invite you to submit a revised version of the manuscript that addresses the points raised during the review process.

Please submit your revised manuscript by Aug 29 2025 11:59PM. If you will need more time than this to complete your revisions, please reply to this message or contact the journal office at plosone@plos.org. When you're ready to submit your revision, log on to https://www.editorialmanager.com/pone/ and select the 'Submissions Needing Revision' folder to locate your manuscript file.

Please include the following items when submitting your revised manuscript:

A rebuttal letter that responds to each point raised by the academic editor and reviewer(s). You should upload this letter as a separate file labeled 'Response to Reviewers'.

A marked-up copy of your manuscript that highlights changes made to the original version. You should upload this as a separate file labeled 'Revised Manuscript with Track Changes'.

An unmarked version of your revised paper without tracked changes. You should upload this as a separate file labeled 'Manuscript'.

If you would like to make changes to your financial disclosure, please include your updated statement in your cover letter. Guidelines for resubmitting your figure files are available below the reviewer comments at the end of this letter.

If applicable, we recommend that you deposit your laboratory protocols in protocols.io to enhance the reproducibility of your results. Protocols.io assigns your protocol its own identifier (DOI) so that it can be cited independently in the future. For instructions see: https://journals.plos.org/plosone/s/submission-guidelines#loc-laboratory-protocols. Additionally, PLOS ONE offers an option for publishing peer-reviewed Lab Protocol articles, which describe protocols hosted on protocols.io. Read more information on sharing protocols at https://plos.org/protocols?utm_medium=editorial-email&utm_source=authorletters&utm_campaign=protocols.

We look forward to receiving your revised manuscript.

Kind regards,

Muhammad Mohsin Khan

Academic Editor

PLOS ONE

Journal requirements:

When submitting your revision, we need you to address these additional requirements.

1. Please ensure that your manuscript meets PLOS ONE's style requirements, including those for file naming. The PLOS ONE style templates can be found at https://journals.plos.org/plosone/s/file?id=wjVg/PLOSOne_formatting_sample_main_body.pdf andhttps://journals.plos.org/plosone/s/file?id=ba62/PLOSOne_formatting_sample_title_authors_affiliations.pdf

**Response:** Thank you for your guidance. We have carefully revised our manuscript to ensure full compliance with PLOS ONE's style requirements.

2. Please ensure that your PRISMA flow diagram is included in your main manuscript file as Figure 1; please see the PLOS ONE submission guidelines for systematic reviews and meta-analyses at <https://journals.plos.org/plosone/s/submission-guidelines#loc-systematic-reviews-and-meta-analyses>.

**Response:** Thank you. We have updated and included the PRISMA flow diagram in the main manuscript file as Figure 1 in page eight, in accordance with PLOS ONE's submission guidelines for systematic reviews and meta-analyses.

3. Please include a caption for figure 1.

**Response:** Thank you for your critical comment regarding Figure 1. We fully acknowledge the importance of securing appropriate licensing for published figures. Although we contacted the original authors (Tomaszewski et al., 2016) to request permission to reuse their illustration, we have not received a response. As a result, to ensure compliance with copyright guidelines, we have removed the original figure. Instead, we have inserted a detailed textual description of the sciatic nerve variations in relation to the piriformis muscle, following the classification by Tomaszewski et al. (2016), in the revised manuscript [lines 49-59]. This approach preserves the clarity and scientific value of the content while avoiding any potential copyright issues.

Additional Editor Comments (if provided):

[Note: HTML markup is below. Please do not edit.]

Reviewers' comments:

Reviewer's Responses to Questions

Comments to the Author

1. Is the manuscript technically sound, and do the data support the conclusions?

The manuscript must describe a technically sound piece of scientific research with data that supports the conclusions. Experiments must have been conducted rigorously, with appropriate controls, replication, and sample sizes. The conclusions must be drawn appropriately based on the data presented.

Reviewer #1: Yes

Reviewer #2: Partly

2. Has the statistical analysis been performed appropriately and rigorously?

Reviewer #1: Yes

Reviewer #2: N/A

3. Have the authors made all data underlying the findings in their manuscript fully available?

The PLOS Data policy requires authors to make all data underlying the findings described in their manuscript fully available without restriction, with rare exception (please refer to the Data Availability Statement in the manuscript PDF file). The data should be provided as part of the manuscript or its supporting information, or deposited to a public repository. For example, in addition to summary statistics, the data points behind means, medians and variance measures should be available. If there are restrictions on publicly sharing data—e.g. participant privacy or use of data from a third party—those must be specified.

Reviewer #1: No

Reviewer #2: Yes

4. Is the manuscript presented in an intelligible fashion and written in standard English?

PLOS ONE does not copyedit accepted manuscripts, so the language in submitted articles must be clear, correct, and unambiguous. Any typographical or grammatical errors should be corrected at revision, so please note any specific errors here.

Reviewer #1: Yes

Reviewer #2: Yes

5. Review Comments to the Author

Please use the space provided to explain your answers to the questions above. You may also include additional comments for the author, including concerns about dual publication, research ethics, or publication ethics. (Please upload your review as an attachment if it exceeds 20,000 characters)

Reviewer #1: The authors performed a systematic review and meta-analysis of studies related to SN anatomy to quantify the pooled prevalence of sciatic nerve variations per racial subgroup. Overall, this study is of interest, as specific racial considerations of SN anatomy have not been thoroughly investigated.

**Response:** The authors thank the reviewer for this kind deep insight. We sincerely appreciate your recognition of the relevance of our study and the novelty of addressing racial variations in sciatic nerve anatomy.

This manuscript is overall well-written. There are a few important points related to methods, results, conclusions, and grammar listed below, to which I would appreciate author's attention to.

Methods:

**Response:** Thank you for your positive assessment of our manuscript and for noting that it is overall well-written. We also appreciate your constructive feedback on the methods, results, conclusions, and grammar. We have carefully reviewed each of your points and made the necessary revisions accordingly. Please see our detailed responses below.

1. Limiting the study to English-language articles may introduce language bias, particularly in a study assessing racial variation. Including non-English studies with translation support would enhance comprehensiveness. Was there a reason only English studies were assessed?

**Response:** We appreciate the reviewer’s important observation regarding potential language bias. In our study, we did not strictly exclude non-English articles; however, we required at least an English abstract to preliminarily assess the article’s relevance. For articles without English full texts but with abstracts indicating eligibility, we attempted to obtain translations when possible. Unfortunately, due to resource limitations and difficulties in accessing reliable translations for certain full texts, especially in less commonly spoken languages, we were unable to include all non-English studies. We acknowledge that this may introduce some degree of language bias and have addressed this point as a limitation in the Discussion section of the manuscript from line 319 to 322.

2. In general, using website search engine like google and Yahoo is atypical in this setting. Inclusion of specialized medical databases (e.g., Embase, Scopus) is recommended.

**Response:** Thank you for this helpful comment. We fully agree that specialized medical databases such as Embase and Scopus provide comprehensive coverage and are ideal for systematic reviews. However, due to limited access as we do not have institutional or personal subscriptions to Embase or Scopus we were unable to include them in our current search strategy. To enhance the completeness of our review, we supplemented our primary database search (PubMed, Hinari, and Google Scholar) with manual cross-referencing of all included studies. This approach enabled us to identify additional relevant articles and minimize the risk of missing key studies. As a result, we included over 20 more studies than previous reviews.

3. Please clarify the date range for included studies— is there no predefined earliest date? What does “from their inception” mean?

**Response:** Thank you for raising this important point. We have clarified the date range in line 79 by adding: “Studies published from the start of each database to June 2024 were included.”

We have also retained the term “inception” in lines 102-103, with a slight rewording, “Moreover, this systematic review and meta-analysis included studies published from the inception of each database up to June 2024.” We would like to note that the term “inception” is widely accepted and frequently used in the context of systematic reviews and meta-analyses. It refers to the earliest date from which each database began indexing records. This terminology is commonly used in high-quality reviews following PRISMA and Cochrane guidelines, making it appropriate and standard in this methodological setting.

3. The “methods” portion of the abstract should mention “a systematic review and meta-analysis was conducted to assess the pooled prevalence of SN variations among racial subgroups." or a variation of this, as it is not written there.

**Response:** Thank you for this helpful suggestion. We agree with your comment and have revised the “Methods” section of the abstract to clearly indicate the study design and its primary objective. The revised sentence now reads. ” Methods: A systematic review and meta-analysis were conducted to assess the pooled prevalence of SN variations among racial subgroups. A comprehensive literature search was performed using PubMed, Google Scholar, Hinari, and additional sources, including cross-referenced articles. Subgroup analyses by region, and country were also conducted using a random-effects model. Heterogeneity was assessed with the Cochrane Q test and the I² statistic”

Results:

1. Is it possible based on the data collected to compare if there are differences in the most common type of sciatic nerves between races/continents? Although the prevalence of variations is of interest, an important point would be a specific variant is more common in certain races/regions

**Response:** Thank you for your insightful comment. We agree that identifying whether specific SN variation types are more common in particular racial or regional groups would be valuable. However, the primary studies included in our review did not consistently report the prevalence of each individual variant stratified by race or continent. As a result, we were unable to perform such a detailed comparative analysis.

2. If one conclusion is that regional differences are important, should subgroup analyses be done by grouping regions together and comparing?

**Response:** Thank you for this view. In our study, we conducted subgroup analyses by continent (Africa, Asia, Europe, and America) and by country, with statistical comparisons based on 95% confidence intervals (CIs). As shown in detailed in the results section and Table 2, the highest prevalence of SN variations was found in Asia (16%), with particularly high rates in East Asian countries such as China (38%) and Japan (30%). While most of the subgroup CIs overlapped, indicating no statistically significant differences between many regions, we observed notable exceptions, such as between East Asian countries and other regions. These differences highlight that regional factors may contribute more significantly to the variation in SN anatomy than racial classifications alone. We agree that further analyses based on more refined regional groupings (e.g., East vs. West Asia) may be valuable and recommend this as an area for future research.

3. Please make all data underlying the findings fully available.

**Response:** We appreciate the reviewer’s concern. In response, we have ensured that all data underlying our findings are fully available in the manuscript. Specifically, we have provided comprehensive details of the search strategy, including the databases searched, keywords used, inclusion/exclusion criteria, and the selection process. Furthermore, AQUA domain scores have been summarized in the respective figures. If additional raw data are needed, we are happy to include them as supplementary material upon request.

Conclusions:

1. The overall conclusion per my understanding is that based on continents and race, there is only a modest difference in prevalence of SN variation. However, this is not reflected in the conclusion in the abstract. This should be amended to be the primary conclusion.

**Response:** Thank you for your valuable comment. We agree with your observation that the overall differences in the prevalence of sciatic nerve variations across racial and continental groups are modest and should be clearly reflected in the abstract conclusion. In response, we have revised the conclusion to emphasize this key finding. The updated conclusion now reads:

"This review revealed only modest and statistically non-significant differences in the prevalence of sciatic nerve variations across broad racial and continental groups. In contrast, substantial variation was observed at the regional level, with particularly high prevalence rates in East Asian countries such as China and Japan. These findings suggest that regional factors contribute more to the observed variations than racial factors."

Figures: 1.Was approval obtained by Tomaszewski et al. [6] to use their picture in Figure 1? Please ensure the proper permissions have been obtained from the original authors.

**Response:** Thank you for raising this important point. Regarding Figure 1, we fully acknowledge the importance of securing appropriate licensing for published figures. Although we contacted the original authors (Tomaszewski et al., 2016) to request permission to reuse their illustration, we have not received a response. As a result, to ensure compliance with copyright guidelines, we have removed the original figure. Instead, we have inserted a detailed textual description of the sciatic nerve variations in relation to the piriformis muscle, following the classification by Tomaszewski et al. (2016), in the revised manuscript [lines 49-59]. This approach preserves the clarity and scientific value of the content while avoiding any potential copyright issues.

Figure 2: This image should be slightly modified to correct the grammar to erase the underlined blue segments, and change the sizing so that ideally the vertically oriented words on the left are not squished.

**Response:** Thank you for your insightful observation regarding the PRISMA figure presentation. After a thorough review, we identified several numerical inaccuracies in our previously reported data, particularly concerning the total number of articles retrieved, duplicates removed, and records screened. We have now made the following corrections:

The previously reported figure of 7,190 articles was inaccurate. The correct total number of records retrieved from the databases was 4,336, comprising:

PubMed: 2,535 records (previously misreported as 126)

Hinari: 1,491 records (previously misreported as 3,033)

Google Scholar: Although over 17,000 records were initially identified, we used the Publish or Perish software to export only the first 500 most relevant records.

After removing 415 duplicate records, 3,921 records remained for title and abstract screening. Of these, 3,588 were excluded based on relevance. We retrieved 133 full-text articles for detailed assessment, all of which were successfully obtained and screened.

Additionally, one study from South Africa and one from Brazil each contributed three individual reports, resulting in 55 reports from 51 articles included in the meta-analysis. Furthermore, 303 articles were identified through cross-referencing, of which 20 met the eligibility criteria. This brought the total number of reports included in the final meta-analysis to 75. These corrections are now reflected in the revised PRISMA flow diagram, which has been renumbered as Figure 1 following the removal of the original Figure 1 for ethical reasons. The corrected retrieval numbers are also documented in S1 Appendix, which details the search strategy and article counts per database. The discrepancies occurred due to the unintentional use of a prior review folder during initial data input. We apologize for this oversight. However, we emphasize that this had no impact on the overall results or conclusions of the meta-analysis, the total number of included reports remains 75, as originally reported. We sincerely appreciate your attention to detail, which helped us make these important clarifications.

Grammar: A thorough grammatical review is needed.

Please review for instances where a period should be used “Filters; English, free abstract text, and date from June, 2024, (S1 Appendix)” vs where a comma should be inserted (Moreover this systematic review and meta-analysis included studies conducted from their inception up to June 2024) or at the end of the “outcomes of interest” paragraph.

**Response:** Thank you for your valuable feedback. We have revised the paragraph to improve clarity and address the concerns raised. Specifically, we corrected the grammar and punctuation, ensured appropriate use of conjunctions and filters, and clarified the search strategy with better structure and flow. We have retained the term “inception” in the sentence referring to the database time range, as it is commonly used in systematic reviews to indicate the beginning of database coverage. However, to avoid ambiguity, we have also clarified this by specifying "From the start of each database to June 2024." The revised paragraph is provided in the main text and supplementary appendix (S1 Appendix).

Other specific grammatical notes:

First paragraph: “however, the variation of the SN is common, likely originating from the CPN and TN branches existing as separate entities during the embryological period” should be “variations of the SN are common….”

Second paragraph: “As updated by Tomaszewski et al., subgroups of SN variations are defined by how these divisions exit relative to the piriformis muscle as follow [6]” should be as “follows:”

Period at the end of “Moreover, these studies focused on anatomical variations and their relationships with the piriformis muscle but did not specifically investigate racial differences, despite significant differences being observed”

**Response:** Thank you for the helpful suggestion. In line with your comment and the input from Reviewer 2, we have revised and condensed the sentence to improve clarity and grammatical accuracy.

“Additionally, 303 articles were searched from websites (google &yahoo) and cross reference” should be “cross-referenced.”

**Response:** Thank you for pointing this out. We have corrected the sentence as suggested.

“And this variation was studies by Tomaszewski et al.[6] and Frideriki P[7].” Should be “studied”

**Response:** Thank you for pointing this out, which has now been amended in the revised discussion.

“Our research reveals that Type A variations are the most commonly observed, considered the normal branching pattern of the SN, where the nerve passes undivided under the PM.” Should be changed to “Our research reveals that Type A variations-- considered the normal branching pattern of the SN, where the nerve passes undivided under the PM-- are the most commonly observed”

**Response:** Thank you. We found your comment is helpful and has now been written clearly as suggested.

“This finding aligns with other review studies conducted by Tomaszewski et al.[6], reported a prevalence of 85.2%, and Frideriki P.[7] , who found it to be 90%.” Should be “This finding aligns with other review studies conducted by Tomaszewski et al.[6], who reported a prevalence of 85.2%, and Frideriki P.[7] , who found it to be 90%.”

Please correct: “About 85-90% of individuals in Clinically Oriented Anatomy [75] and Netter's Atlas of Human Anatomy[76]. 85-88% of Gray's Anatomy [77] have reported Type A variations of SN”

**Response:** Thank you very much for your suggestion. We found your comment is helpful and has now been written clearly as suggested.

Add an “a” to “and it is A relatively common occurrence across all studied racial groups, with only modest differences in prevalence”

**Response:** Thank you for pointing this out. We have revised the sentence accordingly.

Add a space to “Table1”

**Response:** Thank you for the observation. We have corrected it to “Table 1” as suggested.

Remove space from “Over all variation” in Table 1.

**Response:** Thank you for your suggestion. We have corrected “Over all variation” to “Overall variation” in Table 1.

Thank you for your attention to these matters.

Reviewer #2: This manuscript examines racial and regional variations in sciatic nerve anatomy through a systematic review and meta-analysis. The topic is clinically relevant, and the methodology is generally appropriate. However, several areas need clarification and tightening to improve the manuscript's precision and focus.

**Response:** We sincerely thank the reviewer for recognizing the clinical relevance and methodological appropriateness of our study. We appreciate your constructive feedback. In response, we have carefully revised the manuscript to enhance clarity, precision, and focus. Specific improvements have been made to the writing, data presentation, and interpretation, as detailed in our point-by-point responses below.

The introduction is too long and should be condensed. It combines anatomical background, classification systems, and study rationale without clear structure. The distinction between racial and regional differences should be handled more critically. The manuscript presents race as a biological category without discussing its complexity or how it was defined in the included studies. It should be clear whether race was self-identified, assumed based on location, or described by the original authors.

**Response:** Thank you for your valuable comment regarding the conceptual and methodological treatment of race in our study. In response, we have revised the introduction to address the complexity of the race construct and clarify the scope of our investigation. The updated paragraph now reads: "Although the concept of race is both socially and biologically complex, studies have reported clear racial differences between populations, with significant distinctions observed between Chinese and Japanese on one hand, and Europeans and Americans on the other [7]. However, while these studies primarily examined anatomical variations in relation to the piriformis muscle, they did not explicitly investigate racial differences, even though they reported significant disparities among populations. To address this gap in the literature, our meta-analysis aims to systematically retrieve global data from clinical anatomy studies and comprehensively analyze and summarize existing evidence on how race influences sciatic nerve variations, providing insights relevant for clinical procedures and anatomical studies."

To further clarify how race was defined and categorized in our review, we have added the following explanation to the Eligibility Criteria section (lines 104-108): "Furthermore, in this review, racial categorization was extracted based on the description provided in the original articles. Where available, we used self-identified race. If the original authors explicitly described the race of study participants, we reported it as described. In cases where neither of these was specified, racial classification was inferred based on the dominant racial group of the study’s geographic location."

The methods are mostly sound. However, the use of general web searches (Google, Yahoo) as sources for studies is questionable. Either justify their inclusion or remove them from the search strategy. The exclusion of non-English studies also introduces bias, which should be acknowledged more directly.

**Response:** Thank you for your valuable comment regarding the use of general web searches such as Google and Yahoo, as well as language restrictions. We apologize for the initial inclusion of these terms in our search strategy description. Our intention was to refer to manual cross-referencing. Therefore, we have replaced the mention of general web searches with “cross-referencing” and refined the search strategy to focus on established electronic databases only.

Regarding the exclusion of non-English studies, we have explicitly acknowledged this limitation in the Limitations section of the manuscript, emphasizing the potential for language bias and its possible impact on the comprehensiveness of our review from line number 319-322.

The manuscript mentions quality assessment using the AQUA checklist, but no summary of quality scores or findings is included. This information would help readers assess the strength of the included studies.

**Response:** Thank you for your valuable observation. In response, we have now added a concise summary of the AQUA checklist findings under the subsection titled "Risk of Bias Assessment Using AQUA Checklist" (lines 189–196). This section highlights the overall quality trends across the five domains, emphasizing that most studies demonstrated a low risk of bias in objectives, study design, descriptive anatomy, and result reporting. However, methodology characterization was identified as the most inconsistent domain, with nearly 58% of studies rated as high or unclear risk. Additionally, we have incorporated a stacked bar graph (Figure 2) to visually represent the domain-wise risk of bias distribution for greater transparency.

The results show high heterogeneity, with I² values above 90 percent in most analyses. This raises concerns about how comparable the studies are. The authors should address this more explicitly and state whether they considered meta-regression or subgroup analysis to explore the causes of this variability.

**Response:** Thank you for your thoughtful comment regarding the high heterogeneity (I² > 90%) observed in our meta-analyses. As pointed out, such levels of heterogeneity raise concerns about the comparability of the included studies. In line with the recommendation by Henry et al. (2016), who emphasized that high heterogeneity is inherent in anatomical meta-analyses and suggested the use of a random-effects model to account for between-study variability [Henry BM, Tomaszewski KA, Walocha JA. Ann Anat. 2016;205:16–21], we employed a random-effects model for all analyses to enhance the validity of our findings.

To further explore sources of heterogeneity, we conducted subgroup analyses based on race, continent, and country. However, despite these efforts, heterogeneity remained high across most subgroups. This persistent heterogeneity likely reflects intrinsic variations in study designs, dissection protocols, definitions of classifications, and sample characteristics. Furthermore, we have explicitly discussed this issue in the Limitations section of the manuscript.

The very high prevalence found in East Asian populations (38 percent in China, 30 percent in Japan) is striking. The authors should examine whether these figures could reflect differences in methodology, sample selection, or classification rather than anatomical differences alone.

**Response:** We appreciate the reviewer’s insightful comment. Upon careful examination, we found that the studies from East Asian populations employed methodologies, sample selection criteria, and classification systems that were generally consistent with those used in other included studies. Therefore, the high prevalence observed in East Asia is unlikely to be solely attributed to methodological or classification differences. Instead, it may reflect true anatomical variation or population-specific characteristics.

The discussion section repeats findings without offering much interpretation. It should focus more on clinical implications. For example, how should a surgeon modify their technique based on the knowledge of these variations?

**Response:** We sincerely thank the reviewer for this track changing comment. In response, we have made significant modifications to the discussion section, specifically from lines 277 to 303. We added four detailed paragraphs focusing on the clinical relevance of sciatic nerve anatomical variations. These additions elaborate on how knowledge of these variants is essential during surgical procedures such as total hip arthroplasty, hip arthroscopy, and sciatic nerve blocks. We also addressed their role in conditions like piriformis syndrome.

The conclusion that regional factors are more important than race is reasonable, but it should be presented more clearly. The manuscript should also recognize the limitations of racial classification in anatomical research.

**Response:** We appreciate the reviewer’s insightful comment. We have added a detailed acknowledgment of the limitations of racial classification in anatomical research. Specifically, we now clarify that race, when not self-reported, was inferred from author-defined or geographically dominant race, and we note the biological and social complexity of race as a category, which may limit its utility in explaining anatomical variation. These clarifications have been added to lines 311-319 in the revised manuscript.

In summary, this review provides useful pooled data on sciatic nerve variations but needs more critical analysis of its assumptions and findings. Revisions should aim to clarify key concepts, address heterogeneity, and make the conclusions more practical and well-supported.

**Response:** We thank the reviewer for the constructive summary and valuable suggestions. In response, we have undertaken a series of targeted revisions to strengthen the manuscript:

We have clarified key concepts related to racial and regional classifications, and addressed the limitations of using race as a variable in anatomical research (see revised conclusion and methods sections). We have included more critical reflection on the assumptions underlying the classification and interpretation of sciatic nerve variations, particularly regarding study inclusion criteria and population grouping.

To address heterogeneity, we have added subgroup analyses and narrative explanations to better account for sources of variation across studies, including geographical, racial, and Countries differences. The conclusion section has been revised to make our findings more clinically relevant, emphasizing how awareness of these anatomical patterns can aid surgeons, anesthesiologists, and clinicians in improving patient outcomes.

6. PLOS authors have the option to publish the peer review history of their article (what does this mean?). If published, this will include your full peer review and any attached files.

If you choose “no”, your identity will remain anonymous but your review may still be made public.

Do you want your identity to be public for this peer review? For information about this choice, including consent withdrawal, please see our Privacy Policy.

Reviewer #1: No

Reviewer #2: No
