# Supplementary material for: Racial variations in sciatic nerve anatomy: A systematic review and meta-analysis
Source: PLoS One. 2026 Mar 5;21(3):e0344170. doi: 10.1371/journal.pone.0344170 (PMC12962491; doi:10.1371/journal.pone.0344170)
Supplement: Attachment — Submitted filename: Suggestions.docx [file pone.0344170.s006.docx]

The authors have incorporated many earlier concerns, and the manuscript has improved in clarity. The corrections in the graphs are appreciated and the detailed description of the limitations. I have two suggestions:

1) Thank you for clarifying your search methods; Although cross-referencing and Google Scholar were used, the absence of Embase/Scopus remains a limitation. This is acceptable given resource constraints, but the methods could be clearer. Suggestion: Add an explicit sentence noting that “without access to certain subscription databases (e.g., Embase, Scopus), our search may have missed relevant studies, though we mitigated this by cross-referencing extensively.” This will help readers also understand and accept this limitation.

2) The meta-analyses yield I² > 90% in nearly all cases. The current discussion mentions this but still presents pooled prevalence as if they are broadly generalizable. Suggestion: Consider a sensitivity analysis (e.g., removing East Asia) to show how much these values drive overall prevalence.

3) Minor grammatical suggestions for clarity

Abstract

- “Subgroup analyses by region, and country”—remove the comma after “region”
- To improve understanding amongst a broad audience, in the abstract results, I would recommend rearranging to first mention “The Type A pattern, considered normal, had the highest pooled prevalence at 85.7%. Among the variations...”

Introduction:

- Line 73: Would say, “…how race **and region** influence….”
- Line 118: “thorough,” not “through”
- Lin 249: Please change comma locations as sown: “Our research reveals that Type A variations, considered the normal branching pattern of the SN where the nerve passes undivided under the PM, are the most commonly observed”
