# Supplementary material for: Racial variations in sciatic nerve anatomy: A systematic review and meta-analysis
Source: PLoS One. 2026 Mar 5;21(3):e0344170. doi: 10.1371/journal.pone.0344170 (PMC12962491; doi:10.1371/journal.pone.0344170)
Supplement: Attachment — Submitted filename: Response_to_Reviewers_auresp_2.docx [file pone.0344170.s008.docx]

**Rebuttal letter**

Dear editor, and reviewers.

We would like to thank you for giving us the opportunity to submit a second revised version of our manuscript titled “Racial Variations in Sciatic Nerve Anatomy: A Systematic Review and Meta-Analysis”. We are too grateful to you and the reviewers for taking the time and effort to read our manuscript and offer insightful comments. Based on these comments and suggestions, we have made careful modifications to the original manuscript. Your informed and helpful feedback helped us think of potential changes for the current version. We believe the manuscript has improved and hope that it meets your high standards. The authors welcome further constructive comments, if any. Please see below for a point-by-point response to comments and concerns. Comments are shown in black, followed by our answers and responses in dark blue. The changes are shown in the marked copy of the manuscript (track chamges).

Best regards,

**PONE-D-24-41926R1**

Racial Variations in Sciatic Nerve Anatomy: A Systematic Review and Meta-Analysis

PLOS One
Dear Dr. Abdu,
Thank you for submitting your manuscript to PLOS ONE. After careful consideration, we feel that it has merit but does not fully meet PLOS ONE’s publication criteria as it currently stands. Therefore, we invite you to submit a revised version of the manuscript that addresses the points raised during the review process.
Please submit your revised manuscript by Mar 05 2026 11:59PM. If you will need more time than this to complete your revisions, please reply to this message or contact the journal office at [plosone@plos.org](mailto:plosone@plos.org). When you're ready to submit your revision, log on to <https://www.editorialmanager.com/pone/> and select the 'Submissions Needing Revision' folder to locate your manuscript file.
Please include the following items when submitting your revised manuscript:

- A letter that responds to each point raised by the academic editor and reviewer(s). You should upload this letter as a separate file labeled 'Response to Reviewers'.
- A marked-up copy of your manuscript that highlights changes made to the original version. You should upload this as a separate file labeled 'Revised Manuscript with Track Changes'.
- An unmarked version of your revised paper without tracked changes. You should upload this as a separate file labeled 'Manuscript'.

If you would like to make changes to your financial disclosure, please include your updated statement in your cover letter. Guidelines for resubmitting your figure files are available below the reviewer comments at the end of this letter.

If applicable, we recommend that you deposit your laboratory protocols in [protocols.io](http://protocols.io/) to enhance the reproducibility of your results. Protocols.io assigns your protocol its own identifier (DOI) so that it can be cited independently in the future. For instructions see: [https://journals.plos.org/plosone/s/submission-guidelines#loc-laboratory-protocols](https://track.editorialmanager.com/CL0/https:%2F%2Fjournals.plos.org%2Fplosone%2Fs%2Fsubmission-guidelines%23loc-laboratory-protocols/1/010f019bd63fe321-268e5ea4-cd87-4c94-bb91-69d7adbcefe9-000000/FAvLEvvyc90Io25-evt0BV70C-mD4cTuAH-0i2Uji5U=246). Additionally, PLOS ONE offers an option for publishing peer-reviewed Lab Protocol articles, which describe protocols hosted on [protocols.io](http://protocols.io/). Read more information on sharing protocols at [https://plos.org/protocols?utm_medium=editorial-email&utm_source=authorletters&utm_campaign=protocols](https://track.editorialmanager.com/CL0/https:%2F%2Fplos.org%2Fprotocols%3Futm_medium=editorial-email%26utm_source=authorletters%26utm_campaign=protocols/1/010f019bd63fe321-268e5ea4-cd87-4c94-bb91-69d7adbcefe9-000000/aExEKxY7u_NAnoEuqigzl2kZUXOg6w1mIFmY_O1xn-o=246).

We look forward to receiving your revised manuscript.

Kind regards,

Oyelola A. Adegboye, PhD
Academic Editor
PLOS One

Journal Requirements:

If the reviewer comments include a recommendation to cite specific previously published works, please review and evaluate these publications to determine whether they are relevant and should be cited. There is no requirement to cite these works unless the editor has indicated otherwise. 

Additional Editor Comments:

1. Given the time elapsed since the original literature search, it would strengthen the manuscript to update the search to include studies published up to the end of 2025, where feasible, to ensure that the findings reflect the most current evidence base.

**Response:** We thank the editor for the suggestion. In response, we have updated our literature search to include studies published up to the end of 2025. In the initially submitted R1 manuscript, 75 reports were included. During the updated search and careful review, we identified one study that had been published twice at different time points in different journals. To avoid duplication and multiplication bias, we included only the more comprehensive version, reducing the number of reports to 74. With the addition of seven new studies (1-7) identified in the updated search, the total number of included reports increased to 81. These updates are reflected in the revised PRISMA flowchart and throughout the manuscript, ensuring that the findings reflect the most current evidence base. Moreover, to align with best practices for anatomical meta-analyses, as recommended by the anatomy working group, we changed our software from Stata to MetaXL version 5.3 (EpiGear International Pty Ltd., Wilston, Queensland, Australia), which is strongly recommended for multiple pooled analyses. This update is clearly described in the Statistical Analysis subsection of the Methods section (lines 132–141).

2. The meta-analysis reports substantial between-study heterogeneity (high I²). The authors should consider a meta-regression or other exploratory analyses (e.g., subgroup or sensitivity analyses) to examine potential study-level factors contributing to this heterogeneity. Where such analyses are not feasible, a clearer discussion of this limitation would be helpful.

**Response:** We thank the editor again for these important points. As described in results section lines 234–241, we conducted sensitivity analyses and assessed publication bias using the LFK index from Doi plots, which demonstrated that our pooled estimates remain robust despite heterogeneity and as noted by Henry et al. (2016), high heterogeneity is a common and inherent feature of anatomical meta-analyses. While meta-regression was not feasible due to the limited number of studies in some subgroups, the observed heterogeneity is consistent with the variability inherent in anatomical studies.
[Note: HTML markup is below. Please do not edit.]
Reviewers' comments:
Reviewer's Responses to Questions

**Comments to the Author**
1. If the authors have adequately addressed your comments raised in a previous round of review and you feel that this manuscript is now acceptable for publication, you may indicate that here to bypass the “Comments to the Author” section, enter your conflict of interest statement in the “Confidential to Editor” section, and submit your "Accept" recommendation.

Reviewer #1: All comments have been addressed

2. Is the manuscript technically sound, and do the data support the conclusions?

The manuscript must describe a technically sound piece of scientific research with data that supports the conclusions. Experiments must have been conducted rigorously, with appropriate controls, replication, and sample sizes. The conclusions must be drawn appropriately based on the data presented.

Reviewer #1: Yes

3. Has the statistical analysis been performed appropriately and rigorously?

Reviewer #1: Yes

4. Have the authors made all data underlying the findings in their manuscript fully available?

The [PLOS Data policy](http://track.editorialmanager.com/CL0/http:%2F%2Fwww.plosone.org%2Fstatic%2Fpolicies.action%23sharing/1/010f019bd63fe321-268e5ea4-cd87-4c94-bb91-69d7adbcefe9-000000/rv_BqxORv8msgFwmDQLhAO6_R-A5v5-fro0teoOYjQU=246) requires authors to make all data underlying the findings described in their manuscript fully available without restriction, with rare exception (please refer to the Data Availability Statement in the manuscript PDF file). The data should be provided as part of the manuscript or its supporting information, or deposited to a public repository. For example, in addition to summary statistics, the data points behind means, medians and variance measures should be available. If there are restrictions on publicly sharing data—e.g. participant privacy or use of data from a third party—those must be specified.

Reviewer #1: Yes

5. Is the manuscript presented in an intelligible fashion and written in standard English?
PLOS ONE does not copyedit accepted manuscripts, so the language in submitted articles must be clear, correct, and unambiguous. Any typographical or grammatical errors should be corrected at revision, so please note any specific errors here.

Reviewer #1: Yes

6. Review Comments to the Author
Please use the space provided to explain your answers to the questions above. You may also include additional comments for the author, including concerns about dual publication, research ethics, or publication ethics. (Please upload your review as an attachment if it exceeds 20,000 characters)

Reviewer #1: The authors have incorporated many earlier concerns, and the manuscript has improved in clarity. The manuscript is technically sound (data are available, methods are reproducible, ethical criteria are satisfied, and the paper is intelligible). The corrections in the graphs are appreciated and the detailed description of the limitations.

**Response:** We sincerely thank the reviewer for their encouraging comments and positive assessment of our manuscript. We are glad that the clarifications, corrections, and detailed description of limitations have improved the readability and quality of the work. We greatly appreciate the reviewer’s constructive feedback throughout the review process

I have two suggestions:
1) Thank you for clarifying your search methods; Although cross-referencing and Google Scholar were used, the absence of Embase/Scopus remains a limitation. This is acceptable given resource constraints, but the methods could be clearer. Suggestion: Add an explicit sentence noting that “without access to certain subscription databases (e.g., Embase, Scopus), our search may have missed relevant studies, though we mitigated this by cross-referencing extensively.” This will help readers also understand and accept this limitation.

**Response:** We thank the reviewer for this helpful suggestion. We have added a statement to clarify the limitations of our literature search: “Moreover, without access to certain subscription databases (e.g., Embase, Scopus), our search may have missed relevant studies, though we mitigated this by cross-referencing extensively.” This statement has been included in the manuscript in lines 327–329, alongside our explanation of how race was classified, to provide transparency and help readers understand the methods and potential limitations of our study.
2) The meta-analyses yield I² > 90% in nearly all cases. The current discussion mentions this but still presents pooled prevalence as if they are broadly generalizable. Suggestion: Consider a sensitivity analysis (e.g., removing East Asia) to show how much these values drive overall prevalence.

**Response:** We thank the reviewer for this important comment. To address the concern, we conducted sensitivity analyses, including assessing the influence of studies from East Asia on the overall pooled prevalence. These analyses showed that excluding East Asian studies did not significantly alter the overall prevalence or the estimates for Asia. For clarity, we have added a new paragraph in lines 234–241 to present these findings and highlight that the pooled prevalence remains robust despite high heterogeneity.
3) Minor grammatical suggestions for clarity
Abstract
- “Subgroup analyses by region, and country”—remove the comma after “region”

**Response:** We thank the reviewer for the suggestion. The comma after “region” has been removed, and the phrase now reads: “Subgroup analyses by region and country.
- To improve understanding amongst a broad audience, in the abstract results, I would recommend rearranging to first mention “The Type A pattern, considered normal, had the highest pooled prevalence at 85.7%. Among the variations...”

**Response:** We thank the reviewer for this helpful suggestion. In line with the recommendation, the abstract results have been revised to begin with the normal Type A pattern, followed by the variations. We have also updated the prevalence estimates in accordance with the latest analysis. The revised text now reads: Type A, considered the normal pattern, had the highest pooled prevalence at 86%. The remaining 14% represented variations of the sciatic nerve (SN). Among these, Type B was the most common at 7%, followed by Type C and G each observed in 2% of limbs, while less frequent variations included Type Type D (1%), Type E (0%), and Type F (0% (0-1)).
Introduction:
- Line 73: Would say, “…how race and region influence….”

**Response:** We thank the reviewer for the suggestion. The text has been updated to read “…how race and region influence…,” as recommended.
- Line 118: “thorough,” not “through”

**Response:** We thank the reviewer for pointing this out. The text has been corrected to “thorough” in line 121.
- Lin 249: Please change comma locations as sown: “Our research reveals that Type A variations, considered the normal branching pattern of the SN where the nerve passes undivided under the PM, are the most commonly observed”

**Response:** We thank the reviewer for the helpful suggestion. The sentence has been revised for clarity and now reads: “our review reveals that Type A variations, considered the normal branching pattern of the SN where the nerve passes undivided under the PM, are the most commonly observed.” This revised sentence can be found in lines 215–217 of the manuscript.

7. PLOS authors have the option to publish the peer review history of their article ([what does this mean?](https://track.editorialmanager.com/CL0/https:%2F%2Fjournals.plos.org%2Fplosone%2Fs%2Feditorial-and-peer-review-process%23loc-peer-review-history/1/010f019bd63fe321-268e5ea4-cd87-4c94-bb91-69d7adbcefe9-000000/layFsDQ7IU7xH06FKZ1G4qk73kMV6lhGyO9v-a8ENE8=246)). If published, this will include your full peer review and any attached files.
If you choose “no”, your identity will remain anonymous but your review may still be made public.
**Do you want your identity to be public for this peer review?** For information about this choice, including consent withdrawal, please see our [Privacy Policy](https://track.editorialmanager.com/CL0/https:%2F%2Fwww.plos.org%2Fprivacy-policy/1/010f019bd63fe321-268e5ea4-cd87-4c94-bb91-69d7adbcefe9-000000/rh-p7v8Vbg7oBI_ROw6WVCGce--X1Zz6XdMesmlD6o4=246).

Reviewer #1: No

1.	Yadav SK, Yadav R, Adhikari RK. Composite anatomical variations of the sciatic nerve in relation to the piriformis muscle observed in cadaveric studies. Journal of Chitwan Medical College. 2025;15(6):101-7.

2.	Khan GA, Gautam A, Shakya S, Shrestha A. Study of a variation of lumbosacral plexus: sciatic nerve and its clinical significance. Journal of Chitwan Medical College. 2022;12(4):51-4.

3.	Kegoye ES, Ojewale AO, Ezekiel W, Usman IM, Aigbogun EO, Adewale AO, et al. Morphologic and morphometric bilateral analysis and sexual dimorphism in sciatic nerves of adult cadaveric specimens in Uganda. BMC Musculoskeletal Disorders. 2025;26(1):422.

4.	Jyothi J, Praveen V, Sarada T. Anatomical Variations of High Division of Sciatic Nerve and its Relation to Piriformis–A Cadaveric Study. International Journal of Medical and Pharmaceutical Research. 2025;6:1595-600.

5.	Habumuremyi S, Kubwimana O, Archibong V, Gashegu J. Sciatic nerve variations around the piriformis muscle and bifurcation level: An anatomical study. East African Orthopaedic Journal. 2024;18(2):58-64.

6.	Ghosh D, Anandi S, Bandyopadhyay D, Jamwal V. Sciatic Nerve: Morphological Diversification in the Cadavers and Its Clinical Relevance: A Descriptive Observational Study. Journal of Medical Academics. 2025;8(2):95-102.

7.	Amasiatu V, Oyakhire M, Oladipo G. Termination pattern of sciatic nerve in Nigeria: A cadaver dissection study. JIRMEPS. 2017;12(1):1-6.
