# Supplementary material for: Racial variations in sciatic nerve anatomy: A systematic review and meta-analysis
Source: PLoS One. 2026 Mar 5;21(3):e0344170. doi: 10.1371/journal.pone.0344170 (PMC12962491; doi:10.1371/journal.pone.0344170)
Supplement: Peer review history

# Peer review: Decision Letter 0

**DOI:** 10.1371/journal.pone.0344170.r001

**Authors:** Muhammad Mohsin Khan

15 Jul 2025

Dear Dr. Abdu,

Thank you for submitting your manuscript to PLOS ONE. After careful consideration, we feel that it has merit but does not fully meet PLOS ONE’s publication criteria as it currently stands. Therefore, we invite you to submit a revised version of the manuscript that addresses the points raised during the review process.

Please submit your revised manuscript by Aug 29 2025 11:59PM. If you will need more time than this to complete your revisions, please reply to this message or contact the journal office at plosone@plos.org . When you're ready to submit your revision, log on to https://www.editorialmanager.com/pone/ and select the 'Submissions Needing Revision' folder to locate your manuscript file.

- A rebuttal letter that responds to each point raised by the academic editor and reviewer(s). You should upload this letter as a separate file labeled 'Response to Reviewers'.
- A marked-up copy of your manuscript that highlights changes made to the original version. You should upload this as a separate file labeled 'Revised Manuscript with Track Changes'.
- An unmarked version of your revised paper without tracked changes. You should upload this as a separate file labeled 'Manuscript'.

If you would like to make changes to your financial disclosure, please include your updated statement in your cover letter. Guidelines for resubmitting your figure files are available below the reviewer comments at the end of this letter.

If applicable, we recommend that you deposit your laboratory protocols in protocols.io to enhance the reproducibility of your results. Protocols.io assigns your protocol its own identifier (DOI) so that it can be cited independently in the future. For instructions see: https://journals.plos.org/plosone/s/submission-guidelines#loc-laboratory-protocols . Additionally, PLOS ONE offers an option for publishing peer-reviewed Lab Protocol articles, which describe protocols hosted on protocols.io. Read more information on sharing protocols at https://plos.org/protocols?utm_medium=editorial-email&utm_source=authorletters&utm_campaign=protocols .

We look forward to receiving your revised manuscript.

Kind regards,

Muhammad Mohsin Khan

Academic Editor

PLOS ONE

Journal requirements:

When submitting your revision, we need you to address these additional requirements.

1. Please ensure that your manuscript meets PLOS ONE's style requirements, including those for file naming. The PLOS ONE style templates can be found at

https://journals.plos.org/plosone/s/file?id=wjVg/PLOSOne_formatting_sample_main_body.pdf and

https://journals.plos.org/plosone/s/file?id=ba62/PLOSOne_formatting_sample_title_authors_affiliations.pdf

2. Please ensure that your PRISMA flow diagram is included in your main manuscript file as Figure 1; please see the PLOS ONE submission guidelines for systematic reviews and meta-analyses at https://journals.plos.org/plosone/s/submission-guidelines#loc-systematic-reviews-and-meta-analyses .

3. Please include a caption for figure 1.

Additional Editor Comments (if provided):

[Note: HTML markup is below. Please do not edit.]

Reviewers' comments:

Reviewer's Responses to Questions

**Comments to the Author**

1. Is the manuscript technically sound, and do the data support the conclusions?

Reviewer #1: Yes

Reviewer #2: Partly

**********

2. Has the statistical analysis been performed appropriately and rigorously?

Reviewer #1: Yes

Reviewer #2: N/A

**********

3. Have the authors made all data underlying the findings in their manuscript fully available?

The PLOS Data policy

Reviewer #1: No

Reviewer #2: Yes

**********

4. Is the manuscript presented in an intelligible fashion and written in standard English?

Reviewer #1: Yes

Reviewer #2: Yes

**********

Reviewer #1: The authors performed a systematic review and meta-analysis of studies related to SN anatomy to quantify the pooled prevalence of sciatic nerve variations per racial subgroup. Overall, this study is of interest, as specific racial considerations of SN anatomy have not been thoroughly investigated. This manuscript is overall well-written. There are a few important points related to methods, results, conclusions, and grammar listed below, to which I would appreciate author's attention to.

Methods:

1. Limiting the study to English-language articles may introduce language bias, particularly in a study assessing racial variation. Including non-English studies with translation support would enhance comprehensiveness. Was there a reason only English studies were assessed?

2. In general, using website search engine like google and Yahoo is atypical in this setting. Inclusion of specialized medical databases (e.g., Embase, Scopus) is recommended.

3. Please clarify the date range for included studies— is there no predefined earliest date? What does “from their inception” mean?

4. The “methods” portion of the abstract should mention “a systematic review and meta-analysis was conducted to assess the pooled prevalence of SN variations among racial subgroups." or a variation of this, as it is not written there.

Results:

1. Is it possible based on the data collected to compare if there are differences in the most common type of sciatic nerves between races/continents? Although the prevalence of variations is of interest, an important point would be a specific variant is more common in certain races/regions

2. If one conclusion is that regional differences are important, should subgroup analyses be done by grouping regions together and comparing?

3. Please make all data underlying the findings fully available.

Conclusions:

1. The overall conclusion per my understanding is that based on continents and race, there is only a modest difference in prevalence of SN variation. However, this is not reflected in the conclusion in the abstract. This should be amended to be the primary conclusion.

Figures:

1. Was approval obtained by Tomaszewski et al. [6] to use their picture in Figure 1? Please ensure the proper permissions have been obtained from the original authors.

2. Figure 2: This image should be slightly modified to correct the grammar to erase the underlined blue segments, and change the sizing so that ideally the vertically oriented words on the left are not squished.

Grammar: A thorough grammatical review is needed.

Please review for instances where a period should be used “Filters; English, free abstract text, and date from June, 2024, (S1 Appendix)” vs where a comma should be inserted (Moreover this systematic review and meta-analysis included studies conducted from their inception up to June 2024) or at the end of the “outcomes of interest” paragraph.

Other specific grammatical notes:

First paragraph: “however, the variation of the SN is common, likely originating from the CPN and TN branches existing as separate entities during the embryological period” should be “variations of the SN are common….”

Second paragraph: “As updated by Tomaszewski et al., subgroups of SN variations are defined by how these divisions exit relative to the piriformis muscle as follow [6]” should be as “follows:”

Period at the end of “Moreover, these studies focused on anatomical variations and their relationships with the piriformis muscle but did not specifically investigate racial differences, despite significant differences being observed”

“Additionally, 303 articles were searched from websites (google &yahoo) and cross reference” should be “cross-referenced.”

“And this variation was studies by Tomaszewski et al.[6] and Frideriki P[7].” Should be “studied”

“Our research reveals that Type A variations are the most commonly observed, considered the normal branching pattern of the SN, where the nerve passes undivided under the PM.” Should be changed to “Our research reveals that Type A variations-- considered the normal branching pattern of the SN, where the nerve passes undivided under the PM-- are the most commonly observed”

“This finding aligns with other review studies conducted by Tomaszewski et al.[6], reported a prevalence of 85.2%, and Frideriki P.[7] , who found it to be 90%.” Should be “This finding aligns with other review studies conducted by Tomaszewski et al.[6], who reported a prevalence of 85.2%, and Frideriki P.[7] , who found it to be 90%.”

Please correct: “About 85-90% of individuals in Clinically Oriented Anatomy[75] and Netter's Atlas of Human Anatomy[76]. 85-88% of Gray's Anatomy[77] have reported Type A variations of SN”

Add an “a” to “and it is A relatively common occurrence across all studied racial groups, with only modest differences in prevalence”

Add a space to “Table1”

Remove space from “Over all variation” in Table 1.

Thank you for your attention to these matters.

Reviewer #2: This manuscript examines racial and regional variations in sciatic nerve anatomy through a systematic review and meta-analysis. The topic is clinically relevant, and the methodology is generally appropriate. However, several areas need clarification and tightening to improve the manuscript's precision and focus.

The introduction is too long and should be condensed. It combines anatomical background, classification systems, and study rationale without clear structure. The distinction between racial and regional differences should be handled more critically. The manuscript presents race as a biological category without discussing its complexity or how it was defined in the included studies. It should be clear whether race was self-identified, assumed based on location, or described by the original authors.

The methods are mostly sound. However, the use of general web searches (Google, Yahoo) as sources for studies is questionable. Either justify their inclusion or remove them from the search strategy. The exclusion of non-English studies also introduces bias, which should be acknowledged more directly. The manuscript mentions quality assessment using the AQUA checklist, but no summary of quality scores or findings is included. This information would help readers assess the strength of the included studies.

The results show high heterogeneity, with I² values above 90 percent in most analyses. This raises concerns about how comparable the studies are. The authors should address this more explicitly and state whether they considered meta-regression or subgroup analysis to explore the causes of this variability. The very high prevalence found in East Asian populations (38 percent in China, 30 percent in Japan) is striking. The authors should examine whether these figures could reflect differences in methodology, sample selection, or classification rather than anatomical differences alone.

The discussion section repeats findings without offering much interpretation. It should focus more on clinical implications. For example, how should a surgeon modify their technique based on the knowledge of these variations? The conclusion that regional factors are more important than race is reasonable, but it should be presented more clearly. The manuscript should also recognize the limitations of racial classification in anatomical research.

In summary, this review provides useful pooled data on sciatic nerve variations but needs more critical analysis of its assumptions and findings. Revisions should aim to clarify key concepts, address heterogeneity, and make the conclusions more practical and well-supported.

**********

what does this mean? ). If published, this will include your full peer review and any attached files.

If you choose “no”, your identity will remain anonymous but your review may still be made public.

**Do you want your identity to be public for this peer review?** For information about this choice, including consent withdrawal, please see our Privacy Policy

Reviewer #1: No

Reviewer #2: No

**********

[NOTE: If reviewer comments were submitted as an attachment file, they will be attached to this email and accessible via the submission site. Please log into your account, locate the manuscript record, and check for the action link "View Attachments". If this link does not appear, there are no attachment files.]

While revising your submission, please upload your figure files to the Preflight Analysis and Conversion Engine (PACE) digital diagnostic tool, https://pacev2.apexcovantage.com/ . PACE helps ensure that figures meet PLOS requirements. To use PACE, you must first register as a user. Registration is free. Then, login and navigate to the UPLOAD tab, where you will find detailed instructions on how to use the tool. If you encounter any issues or have any questions when using PACE, please email PLOS at figures@plos.org . Please note that Supporting Information files do not need this step.

# Author comment: Author response to Decision Letter 1

**DOI:** 10.1371/journal.pone.0344170.r002

28 Jul 2025

Rebuttal letter

Dear editor, and reviewers.

We would like to thank you for giving us the opportunity to submit a revised version of our manuscript titled “Racial Variations in Sciatic Nerve Anatomy: A Systematic Review and Meta-Analysis”. We are too grateful to you and the reviewers for taking the time and effort to read our manuscript and offer insightful comments. Based on these comments and suggestions, we have made careful modifications to the original manuscript. Your informed and helpful feedback helped us think of potential changes for the current version. We believe the manuscript has improved and hope that it meets your high standards. The authors welcome further constructive comments, if any. Please see below for a point-by-point response to comments and concerns. Comments are shown in black, followed by our answers and responses in dark blue. The changes are shown in the marked copy of the manuscript (highlighted in blue).

Best regards,

Academic Editor’s Comment

Dear Dr. Abdu,

Thank you for submitting your manuscript to PLOS ONE. After careful consideration, we feel that it has merit but does not fully meet PLOS ONE’s publication criteria as it currently stands. Therefore, we invite you to submit a revised version of the manuscript that addresses the points raised during the review process.

Please submit your revised manuscript by Aug 29 2025 11:59PM. If you will need more time than this to complete your revisions, please reply to this message or contact the journal office at plosone@plos.org. When you're ready to submit your revision, log on to https://www.editorialmanager.com/pone/ and select the 'Submissions Needing Revision' folder to locate your manuscript file.

Please include the following items when submitting your revised manuscript:

A rebuttal letter that responds to each point raised by the academic editor and reviewer(s). You should upload this letter as a separate file labeled 'Response to Reviewers'.

A marked-up copy of your manuscript that highlights changes made to the original version. You should upload this as a separate file labeled 'Revised Manuscript with Track Changes'.

An unmarked version of your revised paper without tracked changes. You should upload this as a separate file labeled 'Manuscript'.

If you would like to make changes to your financial disclosure, please include your updated statement in your cover letter. Guidelines for resubmitting your figure files are available below the reviewer comments at the end of this letter.

If applicable, we recommend that you deposit your laboratory protocols in protocols.io to enhance the reproducibility of your results. Protocols.io assigns your protocol its own identifier (DOI) so that it can be cited independently in the future. For instructions see: https://journals.plos.org/plosone/s/submission-guidelines#loc-laboratory-protocols. Additionally, PLOS ONE offers an option for publishing peer-reviewed Lab Protocol articles, which describe protocols hosted on protocols.io. Read more information on sharing protocols at https://plos.org/protocols?utm_medium=editorial-email&utm_source=authorletters&utm_campaign=protocols.

We look forward to receiving your revised manuscript.

Kind regards,

Muhammad Mohsin Khan

Academic Editor

PLOS ONE

Journal requirements:

When submitting your revision, we need you to address these additional requirements.

1. Please ensure that your manuscript meets PLOS ONE's style requirements, including those for file naming. The PLOS ONE style templates can be found at https://journals.plos.org/plosone/s/file?id=wjVg/PLOSOne_formatting_sample_main_body.pdf and https://journals.plos.org/plosone/s/file?id=ba62/PLOSOne_formatting_sample_title_authors_affiliations.pdf

Response: Thank you for your guidance. We have carefully revised our manuscript to ensure full compliance with PLOS ONE's style requirements.

2. Please ensure that your PRISMA flow diagram is included in your main manuscript file as Figure 1; please see the PLOS ONE submission guidelines for systematic reviews and meta-analyses at https://journals.plos.org/plosone/s/submission-guidelines#loc-systematic-reviews-and-meta-analyses.

Response: Thank you. We have updated and included the PRISMA flow diagram in the main manuscript file as Figure 1 in page eight, in accordance with PLOS ONE's submission guidelines for systematic reviews and meta-analyses.

3. Please include a caption for figure 1.

Response: Thank you for your critical comment regarding Figure 1. We fully acknowledge the importance of securing appropriate licensing for published figures. Although we contacted the original authors (Tomaszewski et al., 2016) to request permission to reuse their illustration, we have not received a response. As a result, to ensure compliance with copyright guidelines, we have removed the original figure. Instead, we have inserted a detailed textual description of the sciatic nerve variations in relation to the piriformis muscle, following the classification by Tomaszewski et al. (2016), in the revised manuscript [lines 49-59]. This approach preserves the clarity and scientific value of the content while avoiding any potential copyright issues.

Additional Editor Comments (if provided):

[Note: HTML markup is below. Please do not edit.]

Reviewers' comments:

Reviewer's Responses to Questions

Comments to the Author

1. Is the manuscript technically sound, and do the data support the conclusions?

The manuscript must describe a technically sound piece of scientific research with data that supports the conclusions. Experiments must have been conducted rigorously, with appropriate controls, replication, and sample sizes. The conclusions must be drawn appropriately based on the data presented.

Reviewer #1: Yes

Reviewer #2: Partly

2. Has the statistical analysis been performed appropriately and rigorously?

Reviewer #1: Yes

Reviewer #2: N/A

3. Have the authors made all data underlying the findings in their manuscript fully available?

The PLOS Data policy requires authors to make all data underlying the findings described in their manuscript fully available without restriction, with rare exception (please refer to the Data Availability Statement in the manuscript PDF file). The data should be provided as part of the manuscript or its supporting information, or deposited to a public repository. For example, in addition to summary statistics, the data points behind means, medians and variance measures should be available. If there are restrictions on publicly sharing data—e.g. participant privacy or use of data from a third party—those must be specified.

Reviewer #1: No

Reviewer #2: Yes

4. Is the manuscript presented in an intelligible fashion and written in standard English?

PLOS ONE does not copyedit accepted manuscripts, so the language in submitted articles must be clear, correct, and unambiguous. Any typographical or grammatical errors should be corrected at revision, so please note any specific errors here.

Reviewer #1: Yes

Reviewer #2: Yes

5. Review Comments to the Author

Please use the space provided to explain your answers to the questions above. You may also include additional comments for the author, including concerns about dual publication, research ethics, or publication ethics. (Please upload your review as an attachment if it exceeds 20,000 characters)

Reviewer #1: The authors performed a systematic review and meta-analysis of studies related to SN anatomy to quantify the pooled prevalence of sciatic nerve variations per racial subgroup. Overall, this study is of interest, as specific racial considerations of SN anatomy have not been thoroughly investigated.

Response: The authors thank the reviewer for this kind deep insight. We sincerely appreciate your recognition of the relevance of our study and the novelty of addressing racial variations in sciatic nerve anatomy.

This manuscript is overall well-written. There are a few important points related to methods, results, conclusions, and grammar listed below, to which I would appreciate author's attention to.

Methods:

Response: Thank you for your positive assessment of our manuscript and for noting that it is overall well-written. We also appreciate your constructive feedback on the methods, results, conclusions, and grammar. We have carefully reviewed each of your points and made the necessary revisions accordingly. Please see our detailed responses below.

1. Limiting the study to English-language articles may introduce language bias, particularly in a study assessing racial variation. Including non-English studies with translation support would enhance comprehensiveness. Was there a reason only English studies were assessed?

Response: We appreciate the reviewer’s important observation regarding potential language bias. In our study, we did not strictly exclude non-English articles; however, we required at least an English abstract to preliminarily assess the article’s relevance. For articles without English full texts but with abstracts indicating eligibility, we attempted to obtain translations when possible. Unfortunately, due to resource limitations and difficulties in accessing reliable translations for certain full texts, especially in less commonly spoken languages, we were unable to include all non-English studies. We acknowledge that this may introduce some degree of language bias and have addressed this point as a limitation in the Discussion section of the manuscript from line 319 to 322.

2. In general, using website search engine like google and Yahoo is atypical in this setting. Inclusion of specialized medical databases (e.g., Embase, Scopus) is recommended.

Response: Thank you for this helpful comment. We fully agree that specialized medical databases such as Embase and Scopus provide comprehensive coverage and are ideal for systematic reviews. However, due to limited access as we do not have institutional or personal subscriptions to Embase or Scopus we were unable to include them in our current search strategy. To enhance the completeness of our review, we supplemented our primary database search (PubMed, Hinari, and Google Scholar) with manual cross-referencing of all included studies. This approach enabled us to identify additional relevant articles and minimize the risk of missing key studies. As a result, we included over 20 more studies than previous reviews.

3. Please clarify the date range for included studies— is there no predefined earliest date? What does “from their inception” mean?

Response: Thank you for raising this important point. We have clarified the date range in line 79 by adding: “Studies published from the start of each database to June 2024 were included.”

We have also retained the term “inception” in lines 102-103, with a slight rewording, “Moreover, this systematic review and meta-analysis included studies published from the inception of each database up to June 2024.” We would like to note that the term “inception” is widely accepted and frequently used in the context of systematic reviews and meta-analyses. It refers to the earliest date from which each database began indexing records. This terminology is commonly used in high-quality reviews following PRISMA and Cochrane guidelines, making it appropriate and standard in this methodological setting.

3. The “methods” portion of the abstract should mention “a systematic review and meta-analysis was conducted to assess the pooled prevalence of SN variations among racial subgroups." or a variation of this, as it is not written there.

Response: Thank you for this helpful suggestion. We agree with your comment and have revised the “Methods” section of the abstract to clearly indicate the study design and its primary objective. The revised sentence now reads. ” Methods: A systematic review and meta-analysis were conducted to assess the pooled prevalence of SN variations among racial subgroups. A comprehensive literature search was performed using PubMed, Google Scholar, Hinari, and additional sources, including cross-referenced articles. Subgroup analyses by region, and country were also conducted using a random-effects model. Heterogeneity was assessed with the Cochrane Q test and the I² statistic”

Results:

1. Is it possible based on the data collected to compare if there are differences in the most common type of sciatic nerves between races/continents? Although the prevalence of variations is of interest, an important point would be a specific variant is more common in certain races/regions

Response: Thank you for your insightful comment. We agree that identifying whether specific SN variation types are more common in particular racial or regional groups would be valuable. However, the primary studies included in our review did not consistently report the prevalence of each individual variant stratified by race or continent. As a result, we were unable to perform such a detailed comparative analysis.

2. If one conclusion is that regional differences are important, should subgroup analyses be done by grouping regions together and comparing?

Response: Thank you for this view. In our study, we conducted subgroup analyses by continent (Africa, Asia, Europe, and America) and by country, with statistical comparisons based on 95% confidence intervals (CIs). As shown in detailed in the results section and Table 2, the highest prevalence of SN variations was found in Asia (16%), with particularly high rates in East Asian countries such as China (38%) and Japan (30%). While most of the subgroup CIs overlapped, indicating no statistically significant differences between many regions, we observed notable exceptions, such as between East Asian countries and other regions. These differences highlight that regional factors may contribute more significantly to the variation in SN anatomy than racial classifications alone. We agree that further analyses based on more refined regional groupings (e.g., East vs. West Asia) may be valuable and recommend this as an area for future research.

3. Please make all data underlying the findings fully available.

Response: We appreciate the reviewer’s concern. In response, we have ensured that all data underlying our findings are fully available in the manuscript. Specifically, we have provided comprehensive details of the search strategy, including the databases searched, keywords used, inclusion/exclusion criteria, and the selection process. Furthermore, AQUA domain scores have been summarized in the respective figures. If additional raw data are needed, we are happy to include them as supplementary material upon request.

Conclusions:

1. The overall conclusion per my understanding is that based on continents and race, there is only a modest difference in prevalence of SN variation. However, this is not reflected in the conclusion in the abstract. This should be amended to be the primary conclusion.

Response: Thank you for your valuable comment. We agree with your observation that the overall differences in the prevalence of sciatic nerve variations across racial and continental groups are modest and should be clearly reflected in the abstract conclusion. In response, we have revised the conclusion to emphasize this key finding. The updated conclusion now reads:

"This review revealed only modest and statistically non-significant differences in the prevalence of sciatic nerve variations across broad racial and continental groups. In contrast, substantial variation was observed at the regional level, with particularly high prevalence rates in East Asian countries such as China and Japan. These findings suggest that regional factors contribute more to the observed variations than racial factors."

Figures: 1.Was approval obtained by Tomaszewski et al. [6] to use their picture in Figure 1? Please ensure the proper permissions have been obtained from the original authors.

Response: Thank you for raising this important point. Regarding Figure 1, we fully acknowledge the importance of securing appropriate licensing for published figures. Although we contacted the original authors (Tomaszewski et al., 2016) to request permission to reuse their illustration, we have not received a response. As a result, to ensure compliance with copyright guidelines, we have removed the original figure. Instead, we

Attachment Submitted filename: Response to Reviewers.docx

# Peer review: Decision Letter 1

**DOI:** 10.1371/journal.pone.0344170.r003

**Authors:** Oyelola Adegboye

19 Jan 2026

Dear Dr. Abdu,

Thank you for submitting your manuscript to PLOS ONE. After careful consideration, we feel that it has merit but does not fully meet PLOS ONE’s publication criteria as it currently stands. Therefore, we invite you to submit a revised version of the manuscript that addresses the points raised during the review process.

Please submit your revised manuscript by Mar 05 2026 11:59PM. If you will need more time than this to complete your revisions, please reply to this message or contact the journal office at plosone@plos.org . When you're ready to submit your revision, log on to https://www.editorialmanager.com/pone/ and select the 'Submissions Needing Revision' folder to locate your manuscript file.

- A letter that responds to each point raised by the academic editor and reviewer(s). You should upload this letter as a separate file labeled 'Response to Reviewers'.
- A marked-up copy of your manuscript that highlights changes made to the original version. You should upload this as a separate file labeled 'Revised Manuscript with Track Changes'.
- An unmarked version of your revised paper without tracked changes. You should upload this as a separate file labeled 'Manuscript'.

If you would like to make changes to your financial disclosure, please include your updated statement in your cover letter. Guidelines for resubmitting your figure files are available below the reviewer comments at the end of this letter.

If applicable, we recommend that you deposit your laboratory protocols in protocols.io to enhance the reproducibility of your results. Protocols.io assigns your protocol its own identifier (DOI) so that it can be cited independently in the future. For instructions see: https://journals.plos.org/plosone/s/submission-guidelines#loc-laboratory-protocols . Additionally, PLOS ONE offers an option for publishing peer-reviewed Lab Protocol articles, which describe protocols hosted on protocols.io. Read more information on sharing protocols at https://plos.org/protocols?utm_medium=editorial-email&utm_source=authorletters&utm_campaign=protocols .

We look forward to receiving your revised manuscript.

Kind regards,

Oyelola A. Adegboye, PhD

Academic Editor

PLOS One

Journal Requirements:

If the reviewer comments include a recommendation to cite specific previously published works, please review and evaluate these publications to determine whether they are relevant and should be cited. There is no requirement to cite these works unless the editor has indicated otherwise.

Additional Editor Comments:

1. Given the time elapsed since the original literature search, it would strengthen the manuscript to update the search to include studies published up to the end of 2025, where feasible, to ensure that the findings reflect the most current evidence base.

2. The meta-analysis reports substantial between-study heterogeneity (high I²). The authors should consider a meta-regression or other exploratory analyses (e.g., subgroup or sensitivity analyses) to examine potential study-level factors contributing to this heterogeneity. Where such analyses are not feasible, a clearer discussion of this limitation would be helpful.

[Note: HTML markup is below. Please do not edit.]

Reviewers' comments:

Reviewer's Responses to Questions

**Comments to the Author**

Reviewer #1: All comments have been addressed

**********

2. Is the manuscript technically sound, and do the data support the conclusions?

Reviewer #1: Yes

**********

3. Has the statistical analysis been performed appropriately and rigorously?

Reviewer #1: Yes

**********

4. Have the authors made all data underlying the findings in their manuscript fully available?

The PLOS Data policy

Reviewer #1: Yes

**********

5. Is the manuscript presented in an intelligible fashion and written in standard English?

Reviewer #1: Yes

**********

Reviewer #1: The authors have incorporated many earlier concerns, and the manuscript has improved in clarity. The manuscript is technically sound (data are available, methods are reproducible, ethical criteria are satisfied, and the paper is intelligible). The corrections in the graphs are appreciated and the detailed description of the limitations. I have two suggestions:

1) Thank you for clarifying your search methods; Although cross-referencing and Google Scholar were used, the absence of Embase/Scopus remains a limitation. This is acceptable given resource constraints, but the methods could be clearer. Suggestion: Add an explicit sentence noting that “without access to certain subscription databases (e.g., Embase, Scopus), our search may have missed relevant studies, though we mitigated this by cross-referencing extensively.” This will help readers also understand and accept this limitation.

2) The meta-analyses yield I² > 90% in nearly all cases. The current discussion mentions this but still presents pooled prevalence as if they are broadly generalizable. Suggestion: Consider a sensitivity analysis (e.g., removing East Asia) to show how much these values drive overall prevalence.

3) Minor grammatical suggestions for clarity

Abstract

- “Subgroup analyses by region, and country”—remove the comma after “region”

- To improve understanding amongst a broad audience, in the abstract results, I would recommend rearranging to first mention “The Type A pattern, considered normal, had the highest pooled prevalence at 85.7%. Among the variations...”

Introduction:

- Line 73: Would say, “…how race and region influence….”

- Line 118: “thorough,” not “through”

- Lin 249: Please change comma locations as sown: “Our research reveals that Type A variations, considered the normal branching pattern of the SN where the nerve passes undivided under the PM, are the most commonly observed”

**********

what does this mean? ). If published, this will include your full peer review and any attached files.

If you choose “no”, your identity will remain anonymous but your review may still be made public.

**Do you want your identity to be public for this peer review?** For information about this choice, including consent withdrawal, please see our Privacy Policy

Reviewer #1: No

**********

[NOTE: If reviewer comments were submitted as an attachment file, they will be attached to this email and accessible via the submission site. Please log into your account, locate the manuscript record, and check for the action link "View Attachments". If this link does not appear, there are no attachment files.]

To ensure your figures meet our technical requirements, please review our figure guidelines: https://journals.plos.org/plosone/s/figures

You may also use PLOS’s free figure tool, NAAS, to help you prepare publication quality figures: https://journals.plos.org/plosone/s/figures#loc-tools-for-figure-preparation.

NAAS will assess whether your figures meet our technical requirements by comparing each figure against our figure specifications.

Attachment Submitted filename: Suggestions.docx

# Author comment: Author response to Decision Letter 2

**DOI:** 10.1371/journal.pone.0344170.r004

27 Jan 2026

Rebuttal letter

Dear editor, and reviewers.

We would like to thank you for giving us the opportunity to submit a second revised version of our manuscript titled “Racial Variations in Sciatic Nerve Anatomy: A Systematic Review and Meta-Analysis”. We are too grateful to you and the reviewers for taking the time and effort to read our manuscript and offer insightful comments. Based on these comments and suggestions, we have made careful modifications to the original manuscript. Your informed and helpful feedback helped us think of potential changes for the current version. We believe the manuscript has improved and hope that it meets your high standards. The authors welcome further constructive comments, if any. Please see below for a point-by-point response to comments and concerns. Comments are shown in black, followed by our answers and responses in dark blue. The changes are shown in the marked copy of the manuscript (track chamges).

Best regards,

PONE-D-24-41926R1

Racial Variations in Sciatic Nerve Anatomy: A Systematic Review and Meta-Analysis

PLOS One

Dear Dr. Abdu,

Thank you for submitting your manuscript to PLOS ONE. After careful consideration, we feel that it has merit but does not fully meet PLOS ONE’s publication criteria as it currently stands. Therefore, we invite you to submit a revised version of the manuscript that addresses the points raised during the review process.

Please submit your revised manuscript by Mar 05 2026 11:59PM. If you will need more time than this to complete your revisions, please reply to this message or contact the journal office at plosone@plos.org. When you're ready to submit your revision, log on to https://www.editorialmanager.com/pone/ and select the 'Submissions Needing Revision' folder to locate your manuscript file.

Please include the following items when submitting your revised manuscript:

• A letter that responds to each point raised by the academic editor and reviewer(s). You should upload this letter as a separate file labeled 'Response to Reviewers'.

• A marked-up copy of your manuscript that highlights changes made to the original version. You should upload this as a separate file labeled 'Revised Manuscript with Track Changes'.

• An unmarked version of your revised paper without tracked changes. You should upload this as a separate file labeled 'Manuscript'.

If you would like to make changes to your financial disclosure, please include your updated statement in your cover letter. Guidelines for resubmitting your figure files are available below the reviewer comments at the end of this letter.

If applicable, we recommend that you deposit your laboratory protocols in protocols.io to enhance the reproducibility of your results. Protocols.io assigns your protocol its own identifier (DOI) so that it can be cited independently in the future. For instructions see: https://journals.plos.org/plosone/s/submission-guidelines#loc-laboratory-protocols. Additionally, PLOS ONE offers an option for publishing peer-reviewed Lab Protocol articles, which describe protocols hosted on protocols.io. Read more information on sharing protocols at https://plos.org/protocols?utm_medium=editorial-email&utm_source=authorletters&utm_campaign=protocols.

We look forward to receiving your revised manuscript.

Kind regards,

Oyelola A. Adegboye, PhD

Academic Editor

PLOS One

Journal Requirements:

If the reviewer comments include a recommendation to cite specific previously published works, please review and evaluate these publications to determine whether they are relevant and should be cited. There is no requirement to cite these works unless the editor has indicated otherwise.

Additional Editor Comments:

1. Given the time elapsed since the original literature search, it would strengthen the manuscript to update the search to include studies published up to the end of 2025, where feasible, to ensure that the findings reflect the most current evidence base.

Response: We thank the editor for the suggestion. In response, we have updated our literature search to include studies published up to the end of 2025. In the initially submitted R1 manuscript, 75 reports were included. During the updated search and careful review, we identified one study that had been published twice at different time points in different journals. To avoid duplication and multiplication bias, we included only the more comprehensive version, reducing the number of reports to 74. With the addition of seven new studies (1-7) identified in the updated search, the total number of included reports increased to 81. These updates are reflected in the revised PRISMA flowchart and throughout the manuscript, ensuring that the findings reflect the most current evidence base. Moreover, to align with best practices for anatomical meta-analyses, as recommended by the anatomy working group, we changed our software from Stata to MetaXL version 5.3 (EpiGear International Pty Ltd., Wilston, Queensland, Australia), which is strongly recommended for multiple pooled analyses. This update is clearly described in the Statistical Analysis subsection of the Methods section (lines 132–141).

2. The meta-analysis reports substantial between-study heterogeneity (high I²). The authors should consider a meta-regression or other exploratory analyses (e.g., subgroup or sensitivity analyses) to examine potential study-level factors contributing to this heterogeneity. Where such analyses are not feasible, a clearer discussion of this limitation would be helpful.

Response: We thank the editor again for these important points. As described in results section lines 234–241, we conducted sensitivity analyses and assessed publication bias using the LFK index from Doi plots, which demonstrated that our pooled estimates remain robust despite heterogeneity and as noted by Henry et al. (2016), high heterogeneity is a common and inherent feature of anatomical meta-analyses. While meta-regression was not feasible due to the limited number of studies in some subgroups, the observed heterogeneity is consistent with the variability inherent in anatomical studies.

[Note: HTML markup is below. Please do not edit.]

Reviewers' comments:

Reviewer's Responses to Questions

Comments to the Author

1. If the authors have adequately addressed your comments raised in a previous round of review and you feel that this manuscript is now acceptable for publication, you may indicate that here to bypass the “Comments to the Author” section, enter your conflict of interest statement in the “Confidential to Editor” section, and submit your "Accept" recommendation.

Reviewer #1: All comments have been addressed

________________________________________

2. Is the manuscript technically sound, and do the data support the conclusions?

The manuscript must describe a technically sound piece of scientific research with data that supports the conclusions. Experiments must have been conducted rigorously, with appropriate controls, replication, and sample sizes. The conclusions must be drawn appropriately based on the data presented.

Reviewer #1: Yes

________________________________________

3. Has the statistical analysis been performed appropriately and rigorously?

Reviewer #1: Yes

________________________________________

4. Have the authors made all data underlying the findings in their manuscript fully available?

The PLOS Data policy requires authors to make all data underlying the findings described in their manuscript fully available without restriction, with rare exception (please refer to the Data Availability Statement in the manuscript PDF file). The data should be provided as part of the manuscript or its supporting information, or deposited to a public repository. For example, in addition to summary statistics, the data points behind means, medians and variance measures should be available. If there are restrictions on publicly sharing data—e.g. participant privacy or use of data from a third party—those must be specified.

Reviewer #1: Yes

________________________________________

5. Is the manuscript presented in an intelligible fashion and written in standard English?

PLOS ONE does not copyedit accepted manuscripts, so the language in submitted articles must be clear, correct, and unambiguous. Any typographical or grammatical errors should be corrected at revision, so please note any specific errors here.

Reviewer #1: Yes

________________________________________

6. Review Comments to the Author

Please use the space provided to explain your answers to the questions above. You may also include additional comments for the author, including concerns about dual publication, research ethics, or publication ethics. (Please upload your review as an attachment if it exceeds 20,000 characters)

Reviewer #1: The authors have incorporated many earlier concerns, and the manuscript has improved in clarity. The manuscript is technically sound (data are available, methods are reproducible, ethical criteria are satisfied, and the paper is intelligible). The corrections in the graphs are appreciated and the detailed description of the limitations.

Response: We sincerely thank the reviewer for their encouraging comments and positive assessment of our manuscript. We are glad that the clarifications, corrections, and detailed description of limitations have improved the readability and quality of the work. We greatly appreciate the reviewer’s constructive feedback throughout the review process

I have two suggestions:

1) Thank you for clarifying your search methods; Although cross-referencing and Google Scholar were used, the absence of Embase/Scopus remains a limitation. This is acceptable given resource constraints, but the methods could be clearer. Suggestion: Add an explicit sentence noting that “without access to certain subscription databases (e.g., Embase, Scopus), our search may have missed relevant studies, though we mitigated this by cross-referencing extensively.” This will help readers also understand and accept this limitation.

Response: We thank the reviewer for this helpful suggestion. We have added a statement to clarify the limitations of our literature search: “Moreover, without access to certain subscription databases (e.g., Embase, Scopus), our search may have missed relevant studies, though we mitigated this by cross-referencing extensively.” This statement has been included in the manuscript in lines 327–329, alongside our explanation of how race was classified, to provide transparency and help readers understand the methods and potential limitations of our study.

2) The meta-analyses yield I² > 90% in nearly all cases. The current discussion mentions this but still presents pooled prevalence as if they are broadly generalizable. Suggestion: Consider a sensitivity analysis (e.g., removing East Asia) to show how much these values drive overall prevalence.

Response: We thank the reviewer for this important comment. To address the concern, we conducted sensitivity analyses, including assessing the influence of studies from East Asia on the overall pooled prevalence. These analyses showed that excluding East Asian studies did not significantly alter the overall prevalence or the estimates for Asia. For clarity, we have added a new paragraph in lines 234–241 to present these findings and highlight that the pooled prevalence remains robust despite high heterogeneity.

3) Minor grammatical suggestions for clarity

Abstract

- “Subgroup analyses by region, and country”—remove the comma after “region”

Response: We thank the reviewer for the suggestion. The comma after “region” has been removed, and the phrase now reads: “Subgroup analyses by region and country.

- To improve understanding amongst a broad audience, in the abstract results, I would recommend rearranging to first mention “The Type A pattern, considered normal, had the highest pooled prevalence at 85.7%. Among the variations...”

Response: We thank the reviewer for this helpful suggestion. In line with the recommendation, the abstract results have been revised to begin with the normal Type A pattern, followed by the variations. We have also updated the prevalence estimates in accordance with the latest analysis. The revised text now reads: Type A, considered the normal pattern, had the highest pooled prevalence at 86%. The remaining 14% represented variations of the sciatic nerve (SN). Among these, Type B was the most common at 7%, followed by Type C and G each observed in 2% of limbs, while less frequent variations included Type Type D (1%), Type E (0%), and Type F (0% (0-1)).

Introduction:

- Line 73: Would say, “…how race and region influence….”

Response: We thank the reviewer for the suggestion. The text has been updated to read “…how race and region influence…,” as recommended.

- Line 118: “thorough,” not “through”

Response: We thank the reviewer for pointing this out. The text has been corrected to “thorough” in line 121.

- Lin 249: Please change comma locations as sown: “Our research reveals that Type A variations, considered the normal branching pattern of the SN where the nerve passes undivided under the PM, are the most commonly observed”

Response: We thank the reviewer for the helpful suggestion. The sentence has been revised for clarity and now reads: “our review reveals that Type A variations, considered the normal branching pattern of the SN where the nerve passes undivided under the PM, are the most commonly observed.” This revised sentence can be found in lines 215–217 of the manuscript.

________________________________________

7. PLOS authors have the option to publish the peer review history of their article (what does this mean?). If published, this will include your full peer review and any attached files.

If you choose “no”, your identity will remain anonymous but your review may still be made public.

Do you want your identity to be public for this peer review? For information about this choice, including consent withdrawal, please see our Privacy Policy.

Reviewer #1: No

1. Yadav SK, Yadav R, Adhikari RK. Composite anatomical variations of the sciatic nerve in relation to the piriformis muscle observed in cadaveric studies. Journal of Chitwan Medical College. 2025;15(6):101-7.

2. Khan GA, Gautam A, Shakya S, Shrestha A. Study of a variation of lumbosacral plexus: sciatic nerve and its clinical significance. Journal of Chitwan Medical College. 2022;12(4):51-4.

3. Kegoye ES, Ojewale AO, Ezekiel W, Usman IM, Aigbogun EO, Adewale AO, et al. Morphologic and morphometric bilateral analysis and sexual dimorphism in sciatic nerves of adult cadaveric specimens in Uganda. BMC Musculoskeletal Disorders. 2025;26(1):422.

4. Jyothi J, Praveen V, Sarada T. Anatomical Variations of High Division of Sciatic Nerve and its Relation to Piriformis–A Cadaveric Study. International Journal of Medical and Pharmaceutical Research. 2025;6:1595-600.

5. Habumuremyi S, Kubwimana O, Archibong V, Gashegu J. Sciatic nerve variations around the piriformis muscle and bifurcation level: An anatomical study. East African Orthopaedic Journal. 2024;18(2):58-64.

6. Ghosh D, Anandi S, Bandyopadhyay D, Jamwal V. Sciatic Nerve: Morphological Diversification in the Cadavers and Its Clinical Relevance: A Descriptive Observational Study. Journal of Medical Academics. 2025;8(2):95-102.

7. Amasiatu V, Oyakhire M, Oladipo G. Termination pattern of sciatic nerve in Nigeria: A cadaver dissection study. JIRMEPS. 2017;12(1):1-6.

Attachment Submitted filename: Response_to_Reviewers_auresp_2.docx

# Editor report: Decision Letter 2

**DOI:** 10.1371/journal.pone.0344170.r005

**Authors:** Oyelola Adegboye

18 Feb 2026

Racial Variations in Sciatic Nerve Anatomy: A Systematic Review and Meta-Analysis

PONE-D-24-41926R2

Dear Dr. Abdu,

We’re pleased to inform you that your manuscript has been judged scientifically suitable for publication and will be formally accepted for publication once it meets all outstanding technical requirements.

Within one week, you’ll receive an e-mail detailing the required amendments. When these have been addressed, you’ll receive a formal acceptance letter and your manuscript will be scheduled for publication.

An invoice will be generated when your article is formally accepted. Please note, if your institution has a publishing partnership with PLOS and your article meets the relevant criteria, all or part of your publication costs will be covered. Please make sure your user information is up-to-date by logging into Editorial Manager at Editorial Manager®  and clicking the ‘Update My Information' link at the top of the page. For questions related to billing, please contact billing support .

If your institution or institutions have a press office, please notify them about your upcoming paper to help maximize its impact. If they’ll be preparing press materials, please inform our press team as soon as possible -- no later than 48 hours after receiving the formal acceptance. Your manuscript will remain under strict press embargo until 2 pm Eastern Time on the date of publication. For more information, please contact onepress@plos.org.

Kind regards,

Oyelola A. Adegboye, PhD

Academic Editor

PLOS One

Additional Editor Comments (optional):

Reviewers' comments:

# Editor report: Acceptance letter

**DOI:** 10.1371/journal.pone.0344170.r006

**Authors:** Oyelola Adegboye

PONE-D-24-41926R2

PLOS One

Dear Dr. Abdu,

I'm pleased to inform you that your manuscript has been deemed suitable for publication in PLOS One. Congratulations! Your manuscript is now being handed over to our production team.

At this stage, our production department will prepare your paper for publication. This includes ensuring the following:

* All references, tables, and figures are properly cited

* All relevant supporting information is included in the manuscript submission,

* There are no issues that prevent the paper from being properly typeset

You will receive further instructions from the production team, including instructions on how to review your proof when it is ready. Please keep in mind that we are working through a large volume of accepted articles, so please give us a few days to review your paper and let you know the next and final steps.

Lastly, if your institution or institutions have a press office, please let them know about your upcoming paper now to help maximize its impact. If they'll be preparing press materials, please inform our press team within the next 48 hours. Your manuscript will remain under strict press embargo until 2 pm Eastern Time on the date of publication. For more information, please contact onepress@plos.org.

You will receive an invoice from PLOS for your publication fee after your manuscript has reached the completed accept phase. If you receive an email requesting payment before acceptance or for any other service, this may be a phishing scheme. Learn how to identify phishing emails and protect your accounts at https://explore.plos.org/phishing.

If we can help with anything else, please email us at customercare@plos.org.

Thank you for submitting your work to PLOS ONE and supporting open access.

Kind regards,

PLOS ONE Editorial Office Staff

on behalf of

Assoc Prof Oyelola A. Adegboye

Academic Editor

PLOS One
